# Deep Amplicon Sequencing Reveals Culture-dependent Clonal Selection of *Mycobacterium tuberculosis* in Clinical Samples

**DOI:** 10.1093/gpbjnl/qzae046

**Published:** 2024-06-13

**Authors:** Jiuxin Qu, Wanfei Liu, Shuyan Chen, Chi Wu, Wenjie Lai, Rui Qin, Feidi Ye, Yuanchun Li, Liang Fu, Guofang Deng, Lei Liu, Qiang Lin, Peng Cui

**Affiliations:** Department of Clinical Laboratory, Shenzhen Third People’s Hospital, National Clinical Research Center for Infectious Diseases, The Second Affiliated Hospital of Southern University of Science and Technology, Shenzhen 518114, China; Shenzhen Branch, Guangdong Laboratory for Lingnan Modern Agriculture, Genome Analysis Laboratory of the Ministry of Agriculture and Rural Area, Agricultural Genomics Institute at Shenzhen, Chinese Academy of Agricultural Sciences, Shenzhen 518120, China; Department of Clinical Laboratory, Shenzhen Third People’s Hospital, National Clinical Research Center for Infectious Diseases, The Second Affiliated Hospital of Southern University of Science and Technology, Shenzhen 518114, China; Department of Clinical Laboratory, Shenzhen Third People’s Hospital, National Clinical Research Center for Infectious Diseases, The Second Affiliated Hospital of Southern University of Science and Technology, Shenzhen 518114, China; Department of Clinical Laboratory, Shenzhen Third People’s Hospital, National Clinical Research Center for Infectious Diseases, The Second Affiliated Hospital of Southern University of Science and Technology, Shenzhen 518114, China; Shenzhen Branch, Guangdong Laboratory for Lingnan Modern Agriculture, Genome Analysis Laboratory of the Ministry of Agriculture and Rural Area, Agricultural Genomics Institute at Shenzhen, Chinese Academy of Agricultural Sciences, Shenzhen 518120, China; Department of Clinical Laboratory, Shenzhen Third People’s Hospital, National Clinical Research Center for Infectious Diseases, The Second Affiliated Hospital of Southern University of Science and Technology, Shenzhen 518114, China; Department of Clinical Laboratory, Shenzhen Third People’s Hospital, National Clinical Research Center for Infectious Diseases, The Second Affiliated Hospital of Southern University of Science and Technology, Shenzhen 518114, China; Division Two of Pulmonary Diseases Department, Shenzhen Third People’s Hospital, National Clinical Research Center for Infectious Diseases, The Second Affiliated Hospital of Southern University of Science and Technology, Shenzhen 518114, China; Division Two of Pulmonary Diseases Department, Shenzhen Third People’s Hospital, National Clinical Research Center for Infectious Diseases, The Second Affiliated Hospital of Southern University of Science and Technology, Shenzhen 518114, China; Department of Clinical Laboratory, Shenzhen Third People’s Hospital, National Clinical Research Center for Infectious Diseases, The Second Affiliated Hospital of Southern University of Science and Technology, Shenzhen 518114, China; Shenzhen Branch, Guangdong Laboratory for Lingnan Modern Agriculture, Genome Analysis Laboratory of the Ministry of Agriculture and Rural Area, Agricultural Genomics Institute at Shenzhen, Chinese Academy of Agricultural Sciences, Shenzhen 518120, China; Shenzhen Branch, Guangdong Laboratory for Lingnan Modern Agriculture, Genome Analysis Laboratory of the Ministry of Agriculture and Rural Area, Agricultural Genomics Institute at Shenzhen, Chinese Academy of Agricultural Sciences, Shenzhen 518120, China

**Keywords:** *Mycobacterium tuberculosis*, Deep amplicon sequencing, Drug susceptibility testing, Culture-free, Culture-dependent

## Abstract

The commonly-used drug susceptibility testing (DST) relies on bacterial culture and faces shortcomings such as long turnaround time and clonal/subclonal selection biases. Here, we developed a targeted deep amplicon sequencing (DAS) method directly applied to clinical specimens. In this DAS panel, we examined 941 drug-resistant mutations (DRMs) associated with 20 anti-tuberculosis drugs with only 4 pg of initial DNA input, and reduced the clinical testing time from 20 days to 2 days. A prospective study was conducted using 115 clinical specimens, predominantly positive for the Xpert^®^  *Mycobacterium tuberculosis/*rifampicin (Xpert MTB/RIF) assay, to evaluate DRM detection. DAS was performed on culture-free specimens, while culture-dependent isolates were used for phenotypic DST, DAS, and whole-genome sequencing (WGS). For *in silico* molecular DST, our result based on DAS panel revealed the similar accuracy to three published reports based on WGS. For 82 isolates, application of DAS using the resistance-determining mutation method showed better accuracy (93.03% *vs.* 92.16%), sensitivity (96.10% *vs.* 95.02%), and specificity (91.33% *vs.* 90.62%) than WGS using the Mykrobe software. Compared to culture-dependent WGS, culture-free DAS provides a full picture of sequence variation at the population level, exhibiting in detail the gain-and-loss variants caused by bacterial culture. Our study performs a systematic verification of the advantages of DAS in clinical applications and comprehensively illustrates the discrepancies in *Mycobacterium tuberculosis* before and after culture.

## Introduction

Tuberculosis (TB) has long been a serious global public health concern. Approximately 10 million people worldwide develop TB due to *Mycobacterium tuberculosis* complex (MTBC) infections every year [1]. The World Health Organization (WHO) estimated that 150,359 people with multidrug-resistant/rifampicin-resistant (MDR/RR)-TB were enrolled in treatment in 2020, which was equivalent to approximately one in three people who develop drug-resistant (DR)-TB each year [1]. The high burden of DR-TB incidence requires powerful tools for rapid clinical diagnosis and early detection of heteroresistant MTBC populations directly from clinical specimens. Conventional phenotypic drug susceptibility testing (pDST) requires weeks of bacterial culture, while rapid commercial line probe assays endorsed by the WHO for TB diagnosis and molecular drug susceptibility testing (mDST) only cover a limited number of drug-resistant mutations (DRMs) [2]. Whole-genome sequencing (WGS) of MTBC usually requires successful bacterial culture and can be used for species identification, drug resistance prediction, transmission cluster detection, and discovery of new DRMs [3]. The capture of tilling probes for MTBC has been used for genotyping, drug resistance prediction, and transmission inference to replace culture-based WGS [4–6]; however, this method often limits the high bacterial load and omits minor clones owing to inadequate capture efficiency [5,7]. As a country with a high MDR-TB incidence, China is facing escalating challenges in clinical diagnosis and epidemic prevention [1].

Deep amplicon sequencing (DAS) provides a culture-free method covering many mutations within known DR genes, which overcomes the limitations of the methods mentioned above [8]. Targeted sequencing has recently become an increasingly popular method for the diagnosis and DST of MTBC from sputa [7,9–11], culture isolates [12], acid-fast bacilli smears [13], and formalin-fixed paraffin-embedded tissues [14,15]. Although these assays achieved certain sensitivity and specificity for mDST compared with pDST, some anti-TB drugs were often missed.

Nearly all the aforementioned methods were evaluated using pDST as the gold standard. To date, no study has systematically compared the MTBC sequence differences in clinical specimens before and after culture. However, the discrepancies between them have been proven in several studies. For example, when comparing the Xpert^®^  *Mycobacterium tuberculosis/*rifampicin (Xpert MTB/RIF) test on uncultured sputum specimens with the pDST after culture, rifampicin (RIF) susceptibility discrepancies were observed in 2.09% of patients [16]. By comparing the culture-free molecular bacterial load (MBL) assay with the growth assay on solid media under specific drug treatments, the MBL assay detected colonies that were unculturable in solid culture [17]. Furthermore, a study comparing the DAS on uncultured specimens with the WGS after culture identified 7.8% of discordant variants in 109 clinical specimens [8]. In addition, MTBC clone diversity could be reduced or eliminated during culture, which may distort the final resistance profile and mislead clinical treatment [18].

DAS selects relevant gene regions prior to sequencing, reducing the initial DNA input and minimizing the interference from unrelated DNA. In addition, DAS can be used to compare MTBC sequence differences in clinical specimens before and after culture. Here, we designed a DAS panel for direct mDST on clinical specimens, systematically compared culture-free DAS (cfDAS), culture-dependent DAS (cdDAS), and culture-dependent WGS (cdWGS), and evaluated their accuracy in predicting drug resistance. Compared with existing targeted next-generation sequencing technologies which focus on a limited number of genes (3–18 genes) and drugs (2–13 drugs), our DAS panel encompasses the majority of known DR genes (47 genes) and DRMs (941 DRMs) associated with 20 anti-TB drugs base on a comprehensive manual review of scientific literature. To optimize amplification efficiency, the primers of 128 amplicons were divided into two pooled mixes for multiplex polymerase chain reaction (PCR). We aimed to provide a DAS panel covering most anti-TB drugs for rapid, high-throughput, and culture-free mDST diagnosis, while addressing the MTBC sequence differences in clinical specimens before and after culture.

## Results

### Design of the DAS panel

**Figure 1:**
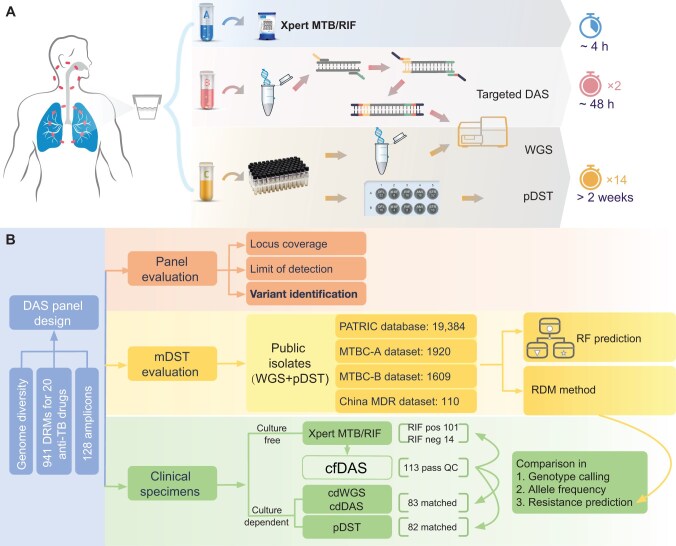
Comparison of DST methods and overview of the DAS data analysis workflow **A**. Comparison of workflows for Xpert MTB/RIF, targeted DAS, WGS, and pDST assays. **B**. Overview of the DAS data analysis workflow. DST, drug susceptibility testing; DAS, deep amplicon sequencing; Xpert MTB/RIF, Xpert^®^  *Mycobacterium tuberculosis*/rifampicin; DRM, drug-resistant mutation; MTB, *Mycobacterium tuberculosis*; MTBC, *Mycobacterium tuberculosis* complex; PATRIC, Pathosystems Resource Integration Center; mDST, molecular DST; WGS, whole-genome sequencing; pDST, phenotypic DST; TB, tuberculosis; MDR, multidrug-resistant; RF, random forest; RIF, rifampicin; pos, positive; neg, negative; cfDAS, culture-free DAS; QC, quality control; cdWGS, culture-dependent WGS; cdDAS, culture-dependent DAS; RDM, resistance-determining mutation.

We designed a DAS panel targeting 941 DRMs from 47 genes associated with 20 anti-TB drugs (Figure S1; **Table 1**, Table S1). The entire workflow of the DAS panel on clinical specimens takes only 48 h, which significantly shortens the turnover time (**Figure 1**A). An overview of the DAS data analysis workflow is shown in Figure 1B.

**Table 1:** DR genes in MTBC targeted by the DAS panel

WHO category	Drug name	Abbreviated drug name	Gene (No. of DRMs)
First-line	Rifampicin	RIF	*rpoB* (68), *rpoC* (2)
	Rifabutin	RFB	*rpoB* (12)
	Isoniazid	INH	*ahpC* (16), *fabD* (1), *fabG1* (11), *furA* (3), *inhA* (15), *iniA* (2), *iniB* (7), *iniC* (3), *kasA* (6), *katG* (89), *nat* (2), *ndh* (4), *Rv0340* (1), *Rv1592c* (1)
	Pyrazinamide	PZA	*panD* (13), *pncA* (172), *rpsA* (3)
	Ethambutol	EMB	*embA* (8), *embB* (28), *embC* (1), *embR* (2), *ubiA* (16)
Group A	Levofloxacin	LFX	*gyrA* (12), *gyrB* (17)
	Moxifloxacin	MFX	*gyrA* (10), *gyrB* (2)
	Bedaquiline	BDQ	*atpE* (9), *Rv0678* (46)
	Linezolid	LZD	*rplC* (3), *rrl* (7)
Group B	Clofazimine	CFZ	*pepQ* (1), *mmpR* (73), *Rv1979c* (1)
	Cycloserine	CS	*alr* (1)
Group C	Delamanid	DLM	*ddn* (2)
	Pretomanid	PMD	*ddn* (2), *fbiC* (3)
	Amikacin	AK	*gidB* (5), *rrs* (4), *whiB7* (6)
	Streptomycin	SM	*gidB* (48), *rpsL* (7), *rrs* (8)
	Ethionamide	ETO	*ethA* (28), *ethR* (1), *fabG1* (3), *inhA* (6)
	Protionamide	PTO	*ethA* (28), *inhA* (4)
	Paraaminosalicylic acid	PAS	*dfrA* (2), *folC* (29), *ribD* (1), *thyA* (37)
Others	Caperomycin	CM	*gidB* (6), *rrs* (4), *tlyA* (11)
	Kanamycin	KM	*eis* (10), *gidB* (6), *rrs* (4), *whiB7* (6)

*Note*: DR, drug-resistant; MTBC, *Mycobacterium tuberculosis* complex; DAS, deep amplicon sequencing; WHO, World Health Organization; DRM, drug-resistant mutation.

**Figure 2:**
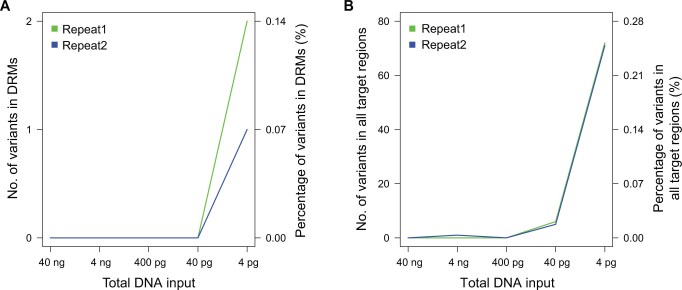
Evaluation of detection capability of the DAS panel **A**. Number of variants in DRMs. **B**. Number of variants in all target regions. The DAS data from the serially diluted genomic DNA samples were compared to the WGS data from the initial DNA sample. Repeat1 and Repeat2 indicate the two replicates of the gradient-dilution assay.

Three rounds of DAS were carried out for the improvements of amplification efficiency and detection capability as well as amplification optimization, and our panel achieved 30× sequencing coverage for 99.37%–100% of the target regions using a DNA mixture of host and bacteria (1 μg host DNA + 200 pg/100 pg/50 pg MTBC DNA) (Figure S2; Table S2). The panel’s detection limit was determined using gradient-diluted DNA samples extracted from one cultured isolate, and our panel efficiently detected 100% of the target regions with 30× sequencing coverage at a minimum concentration of 0.4 pg/μl MTBC genomic DNA (gDNA) in a 10-μl volume (Table S3). Comparative analysis between DAS data from the gradient-diluted DNA samples and WGS data of the initial gDNA sample revealed high consistency (≥ 99.75%) between the two methods, with only a few low-frequency variants [median: 12.87%; interquartile range (IQR): 11.25%–16.18%] identified by DAS as the DNA amount decreased (**Figure 2**; Table S4). When focusing solely on DRMs, one (0.07%) and two (0.14%) variants were identified from two sets of DAS data from the 4-pg DNA samples.

### Evaluation of drug resistance prediction

We collected the whole-genome sequence data as well as the pDST data of 19,384 MTBC isolates from the Pathosystems Resource Integration Center (PATRIC) database. Using the resistance-determining mutation (RDM) method, we predicted the drug resistance of these MTBC isolates to 14 drugs based on the genome sequences at our panel region and compared the *in silico* predictions to the pDST data. The overall prediction accuracy of our DAS panel was 94.47% (sensitivity: 90.40%; specificity: 95.71%) for all 14 drugs (Tables S5–S7). Moreover, another three public datasets were recruited [19,20]. The overall accuracies of our DAS panel combined with the RDM method and the initial WGS-based report were 97.73% (sensitivity: 92.68%; specificity: 98.18%) and 97.37% (sensitivity: 81.45%; specificity: 98.80%) for MTBC_A data (1920 samples, 9 drugs) (Tables S8–S11), 97.07% (sensitivity: 91.14%; specificity: 98.08%) and 96.20% (sensitivity: 82.64%; specificity: 98.52%) for MTBC_B data (1609 samples, 9 drugs) (Tables S12–S15), and 91.14% (sensitivity: 89.52%; specificity: 92.23%) and 91.43% (sensitivity: 91.30%; specificity: 91.52%) for WGS_110 data (110 samples, 13 drugs) (Tables S16–S19). These results suggest that the prediction of drug resistance based on the DAS panel is highly consistent with that based on the whole genome.

Machine learning models enhance the performance of resistance prediction when dealing with large datasets [21]. We constructed classification models for 14 drugs with random forest (RF) to predict drug resistance in our panel region using PATRIC dataset for training. This method achieved an accuracy of 95.10% (sensitivity: 90.04%; specificity: 96.64%), equivalent or better for all 14 drugs compared to our RDM method (accuracy increased by 0%–12.96% with an IQR of 0.25%–1.80%) (Tables S20 and S21). This method also performed well with the other three datasets, with accuracies of 97.61% (sensitivity: 91.25%; specificity: 98.18%) for MTBC_A data (Tables S22 and S23), 97.08% (sensitivity: 90.55%; specificity: 98.20%) for MTBC_B data (Tables S24 and S25), and 89.24% (sensitivity: 84.20%; specificity: 92.92%) for WGS_110 data (12 drugs excluding clofazimine) (Tables S26 and S27). These results indicate that the RF-based classification method performed comparable to the RDM method for drug resistance prediction.

We also used our newly obtained data to evaluate the accuracy of our panel for drug resistance prediction. This analysis included 82 cultured MTBC isolates with pDST and WGS data. The mDST results predicted by the panel-based RDM and RF methods and the WGS-based Mykorbe method were compared with the pDST results (see File S1 for Appendix result 1) (Tables S28–S31). The overall accuracies of drug resistance prediction of our panel for seven drugs were 93.03% (sensitivity: 96.10%; specificity: 91.33%) and 91.99% (sensitivity: 92.54%; specificity: 91.69%) using the RDM and RF methods, respectively (Tables S32 and S33). In contrast, the overall accuracy of drug resistance prediction of the WGS data was 92.16% (sensitivity: 95.02%; specificity: 90.62%) using the Mykrobe method (Table S34). These results suggest that the performance of the panel-based and the WGS-based prediction methods is highly consistent. The discrepancy in resistance prediction among the RDM, Mykrobe, and RF methods is caused by RDM selection for the RDM and Mykrobe methods or limitation of prior information used in the RF method (Figure S3; **Table 2**). Machine learning methods often lack interpretability of classification and thus miss the relationships between mutations and classification. Therefore, we used the known DRMs (the RDM method) for mDST prediction in downstream panel-related analysis.

**Table 2:** Difference among RDM, Mykrobe, and RF methods for drug resistance prediction using cdWGS data from 82 MTBC isolates

Drug	Mutation	RDM	Mykrobe	RF	OR in PATRIC data (95% CI)
INH	*ahpC*:−52C/T	Yes	No	High probability	∞ (7.26–∞)
INH	*katG*:N138H	Yes	No	High probability	19.75 (2.74–862.03)
MFX	*gyrA*:D94A	Yes	Yes	Low probability	13.46 (8.09–23.17)
MFX	*gyrA*:S91P	Yes	Yes	Low probability	7.19 (3.90–13.86)
RIF	*rpoB*:L430P	Yes	Yes	Low probability	4.03 (2.60–6.31)
RIF	*rpoB*:S431T	Yes	No	Low probability	NA
RIF	*rpoB*:H445N	Yes	Yes	Low probability	2.78 (1.58–4.86)
SM	*gidB*:G37E	No	No	High probability	∞ (0.52–∞)

*Note*: RDM, resistance-determining mutation; RF, random forest; cdWGS, culture-dependent whole-genome sequencing; OR, odds ratio; PATRIC, Pathosystems Resource Integration Center; CI, confidence interval; NA, not available.

### Comparison of cfDAS and cdWGS

**Figure 3:**
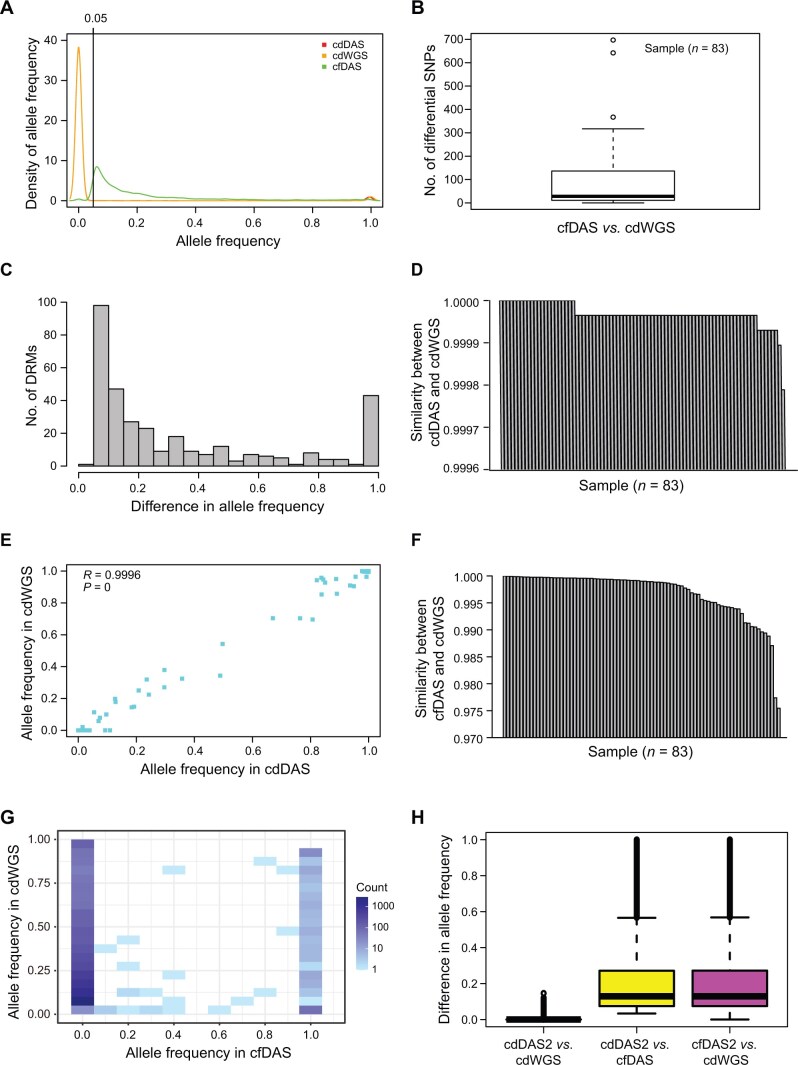
Variant comparison in 83 clinical samples among cdDAS, cdWGS, and cfDAS data **A**. Allele frequency distribution in cdDAS, cdWGS, and cfDAS data (bandwidth = 0.01). **B**. Number of different loci between cfDAS and cdWGS data. **C**. Distribution of DRMs with different allele frequencies between cfDAS and cdWGS data. **D**. Similarity between cdDAS and cdWGS data based on target loci. **E**. Correlation of allele frequencies between cdDAS and cdWGS data. **F**. Similarity between cfDAS and cdWGS data based on target loci. **G**. Correlation of allele frequencies between cfDAS and cdWGS data. **H**. Box plot showing the differences in allele frequency between any two of cfDAS, cdDAS, and cdWGS datasets.

A total of 83 clinical specimens were collected and subjected to cfDAS and cdWGS separately (see File S1 for Appendix result 2). Unexpectedly, a large proportion (88.44%, 8013/9060) of variants identified by cfDAS were heterozygous (**Figure 3**A). In contrast, most variants (91.15%, 1122/1231) detected by cdWGS were homozygous. Further analysis revealed that a significant proportion of the variants identified by cdWGS (93.26%, 1148/1231) were also detected by cfDAS and 81.01% (930/1148) of them were homozygous. This observation suggests that the majority of variants identified by cdWGS are derived from clinical specimens prior to culture. Moreover, totally 27 of the 83 specimens were recognized as mixed infection specimens in cfDAS, and these mixed infection specimens accounted for a significant proportion of the cfDAS-specific variants (80.12%, 6339/7912). We hypothesize that cfDAS can detect sequence variation profiles at the population level, whereas cdWGS is limited to a single or few clones. The discrepancy may be attributed to bacterial culture. Previous studies have reported that bacterial cultures tend to screen out some TB clones, thereby reducing heterogeneity [22].

We compared the single nucleotide polymorphisms (SNPs) detected by cfDAS and cdWGS, and identified 7336 SNPs with difference in allele frequency (AF) using a minimum cutoff of 5% AF change, and 98.17% (7202/7336) of them were different for SNP calling using a minimum cutoff of 5% AF across all 83 specimens (Figure 3B; Table S35). Among these SNPs, 333 SNPs (with 256 being different for SNP calling) from 49 specimens (47 specimens) were DRMs that were linked to 16 drugs. The AF differences for these SNPs ranged from 5% to 100% (Figure 3C). When 10% AF was used as a cutoff for SNP calling, 249 DR SNPs showed AF differences. Among them, 69.88% (174/249) were different for SNP calling in all 83 specimens, which can affect drug resistance prediction. These results indicate significant differences between cfDAS and cdWGS in AF and SNP calling, especially for DRMs, which may further lead to inconsistencies in drug resistance prediction. Among the 7336 SNPs, 97.21% had decreased AFs, while only 2.79% had increased AFs during culture. A similar pattern was observed for the DRMs, with 66.37% showing AF decrease and 33.63% showing AF increase during culture. These results suggest that culture causes clonal selection, thus narrowing down the diversity of MTBC clones, which may lead to a bias in resistance prediction. Notably, 54.64% mutations with higher AFs in the cultured isolates were DRMs, while only 2.06% mutations with higher AFs in the initial specimens were related to drug resistance. This indicates that DR clones may be preferentially selected during culture (*P* = 5.30E−265, Chi-square test). DRMs in culture may be associated with compensatory mutations to fully or partially restore fitness loss. With the limited compensatory mutations in our panel [23], ten cultured isolates had higher AFs in the compensatory mutations in the *rpoC* gene, and seven of them had at least one RIF resistance mutation with the tendency of elevated AF during culture. With RIF resistance mutations as the major allele in the initial specimens, three exceptions were observed in the cultured isolates. We conclude that the inconsistencies between cfDAS and cdWGS are mainly caused by bacterial culture.

To clarify bias raising from the low depth of WGS, we performed DAS on 83 cultured isolates, and found that 90.40% (1120/1239) of the variants were homozygous, showing a similar pattern to that observed in cdWGS (see File S1 for Appendix result 2). Moreover, 99.85% of the variants with different AFs between cfDAS and cdWGS had consistent AFs between cdDAS and cdWGS (< 5% AF difference). Only 14 SNPs showed a minimal 5% AF difference for the target region. Furthermore, the mean pairwise concordance similarity between cdDAS and cdWGS data was 99.9963% [confidence interval (CI): 99.9933%–99.9994%] at target loci (Figure 3D) and 99.8739% (CI: 99.8031%–99.9446%) for AFs. Moreover, a strong relationship was observed between AFs in cdDAS and cdWGS data (*R* = 0.9996, *P* = 0, Pearson’s correlation test) (Figure 3E). These results show a very high degree of consistency in variant identification between cdDAS and cdWGS. In contrast, significant differences were observed in the mean pairwise concordance similarity at target loci (Figure 3F) and AFs, as well as in the correlation of AFs between cfDAS and cdWGS data (Figure 3G). Further, we compared the differences in AF between any two of the three datasets (cfDAS, cdDAS, and cdWGS). Most loci were consistent between culture-dependent datasets (cdDAS *vs.* cdWGS) but inconsistent between culture-free and culture-dependent datasets (cfDAS *vs.* cdDAS/cdWGS) (Figure 3H). These results further illustrate that the inconsistencies between cfDAS and cdWGS are caused by bacterial culture independent of the sequencing method.

### Comparison of mDST with pDST

**Figure 4:**
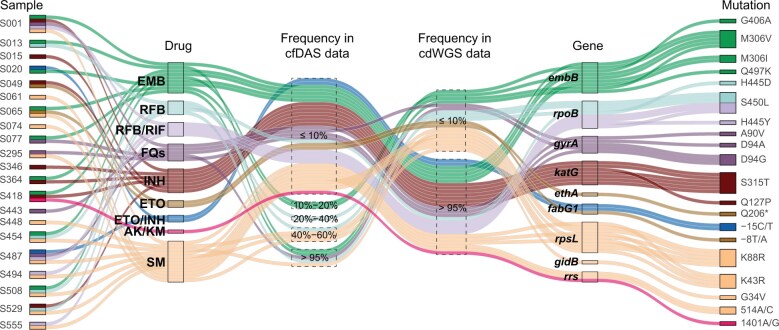
DRMs affecting mDST on clinical specimens before and after culture The alluvial plot generated by the easyalluvial package in R was used to visualize the connection for mutations. The mutations were clustered by sample, drug, frequency in cfDAS data, frequency in cdWGS data, gene, and mutation. Different colors stand for different drugs. EMB, ethambutol; RFB, rifabutin; FQ, fluoroquinolone; INH, isoniazid; ETO, ethionamide; AK, amikacin; KM, kanamycin; SM, streptomycin.

**Figure 5:**
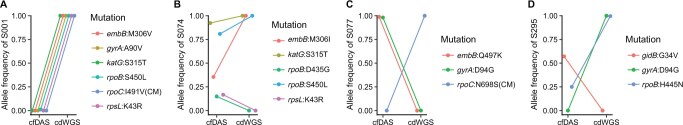
Allele frequency change between cfDAS and cdWGS data **A**. Allele frequency of DRMs in S001 (TBX1) increased in cdWGS data. **B**. Allele frequency of DRMs in S074 (TBX74) increased or decreased in cdWGS data. **C**. Allele frequency of DRMs in S077 (TBX77) decreased in cdWGS data. **D**. Allele frequency of *gyrA*:D94G in S295 (TBX295) increased together with the FQ treatment in cdWGS data. CM, compensatory mutation.

Based on DRMs identified by cdWGS and cfDAS, we performed mDST for 82 specimens using the RDM method (Tables S29 and S36) and compared the results with the corresponding pDST data of ten drugs. The overall accuracy of drug resistance prediction using cfDAS was 86.83% (sensitivity: 82.84%; specificity: 88.77%) (**Table 3**; Table S37), which was slightly lower than that of cdWGS and cdDAS, with an overall accuracy of 92.07% (sensitivity: 94.40%; specificity: 90.94%) for cdWGS (**Table 4**; Table S32) and an overall accuracy of 91.83% (sensitivity: 94.40%; specificity: 90.58%) for cdDAS (Tables S38 and S39). Besides, we compared the mDST results from cfDAS with that from cdWGS, revealing an overall accuracy of 92.80% (sensitivity: 87.13%; specificity: 96.13%) (Table 3). We further found that the difference between cfDAS and cdWGS was mainly caused by the inconsistency in AF. Of the 284 DRMs detected in these 82 specimens, 76 (26.76%) mutations caused genotype differences and led to a 6.10% discrepancy of mDST (**Figure 4**), while 66 (23.24%) mutations had the same genotype but different AFs. For example, specimen S001 was classified as pan-susceptible based on mDST using cfDAS data, whereas it was identified as pre-extensive drug-resistant based on mDST using cdWGS data (**Figure 5**A). Another example was specimen S074, which contained mutations with different AFs and genotypes in the abovementioned datasets (Figure 5B) (see File S1 for Appendix result 3).

**Table 3:** Performance comparison of cfDAS-based mDST with pDST and cdWGS-based mDST on 82 clinical specimens

Drug	pDST resistant		pDST susceptible		cfDAS-based mDST *vs*. pDST (%)				cdWGS-based mDST resistant		cdWGS-based mDST susceptible		cfDAS-based mDST *vs*. cdWGS-based mDST (%)				RMs in FP samples of cfDAS-based mDST *vs.* pDST (sample number)
	TP	FN	FP	TN	Sen	Spe	Acc	PPV	TP	FN	FP	TN	Sen	Spe	Acc	PPV	
RFP	58	4	7	13	93.55	65.00	86.59	89.23	65	4	0	13	94.20	100.0	95.12	100.0	*rpoB*:S450L (2), *rpoB*:D435V (1), *rpoB*:D435G (1), *rpoB*:L452P (1), *rpoB:*S450L–D435V (1), *rpoB*:S450L–D435G (1)
RFB	32	10	13	27	76.19	67.50	71.95	71.11	42	5	3	32	89.36	91.43	90.24	93.33	*rpoB*:S450L (11), *rpoB*:H445Y (1), *rpoB*:H445D (1)
INH	43	10	4	25	81.13	86.21	82.93	91.49	47	8	0	27	85.45	100.0	90.24	100.0	*fabG1*:−15C/T (2), *ahpC*:−52C/T (1), *katG*:S315T (1)
EMB	21	4	9	48	84.00	84.21	84.15	70.00	26	7	4	45	78.79	91.84	86.59	86.67	*embB*:M306I (1), *embB*:M306V (4), *embB*:G406A (1), *embB*:D354A (1), *embB*:Q497K (1), *embB*:M306I–D1024N (1)
LFX	14	4	4	60	77.78	93.75	90.24	77.78	16	3	2	61	84.21	96.83	93.90	88.89	*gyrA*:D94A (2), *gyrA*:D94G (1), *gyrA*:A90V–*gyrB*:D461N (1)
MFX	12	5	6	59	70.59	90.77	86.59	66.67	16	3	2	61	84.21	96.83	93.90	88.89	*gyrA*:D94A (3), *gyrA*:D94G (2), *gyrA*:A90V–*gyrB*:D461N (1)
AK	5	1	0	76	83.33	100.0	98.78	100.0	5	1	0	76	83.33	100.0	98.78	100.0	NA
ETO	2	1	6	73	66.67	92.41	91.46	25.00	6	2	2	72	75.00	97.30	95.12	75.00	*fabG1*:−15C/T (3), *fabG1*:−8T/A (2), *ethA*:Q206* (1)
KM	6	1	0	75	85.71	100.0	98.78	100.0	6	1	0	75	85.71	100.0	98.78	100.0	NA
SM	29	6	13	34	82.86	72.34	76.83	69.05	35	5	7	35	87.50	83.33	85.37	83.33	*rpsL*:K43R (4), *rpsL*:K88R (3), *rpsL*:K43R–K88R (3), *rrs*:517C/T (1), *gidB*:G34V (1), *rpsL*:K43R–*rrs*:514A/C (1)
Total	222	46	62	490	82.84	88.77	86.83	78.17	264	39	20	497	87.13	96.13	92.80	92.96	

*Note*: cfDAS, culture-free deep amplicon sequencing; mDST, molecular drug sensitivity test; pDST, phenotypic drug sensitivity test; TP, true positive; FN, false negative; FP, false positive; TN, true negative; Sen, sensitivity; Spe, specificity; Acc, accuracy; PPV, positive predictive value; RM, resistance-conferring mutation.

**Table 4:** Performance comparison of cdWGS-based mDST with pDST and pDST+RMs on 82 clinical specimens

Drug	pDST resistant		pDST susceptible		cdWGS-based mDST *vs*. pDST (%)				pDST+RMs resistant		pDST+RMs susceptible		cdWGS-based mDST *vs*. pDST+RMs (%)				RMs in FP samples of cdWGS-based mDST *vs*. pDST (sample number)
	TP	FN	FP	TN	Sen	Spe	Acc	PPV	TP	FN	FP	TN	Sen	Spe	Acc	PPV	
RFP	62	0	7	13	100.0	65.00	91.46	89.86	69	0	0	13	100.0	100.0	100.0	100.0	*rpoB*:S450L (2), *rpoB*:D435V (1), *rpoB*:D435G (2), *rpoB*:L452P (1), *rpoB*:S450L–*rpoC*:L516P (1)
RFB	36	6	11	29	85.71	72.50	79.27	76.60	47	6	0	29	88.68	100.0	92.68	100.0	*rpoB*:S450L (10), *rpoB*:H445Y (1)
INH	50	3	5	24	94.34	82.76	90.24	90.91	55	3	0	24	94.83	100.0	96.34	100.0	*fabG1*:−15C/T (2), *ahpC*:−52C/T (1), *katG*:S315T (2)
EMB	25	0	8	49	100.0	85.96	90.24	75.76	33	0	0	49	100.0	100.0	100.0	100.0	*embB*:M306I (4), *embB*:M306V (1), *embB*:M306L (1), *embB*:G406A (1), *embB*:D354A (1)
LFX	17	1	2	62	94.44	96.88	96.34	89.47	19	1	0	62	95.00	100.0	98.78	100.0	*gyrA*:D94A (1), *gyrA*:A90V–*gyrB*:D461N (1)
MFX	15	2	4	61	88.24	93.85	92.68	78.95	19	2	0	61	90.48	100.0	97.56	100.0	*gyrA*:D94A (2), *gyrA*:A90V–D94G (1), *gyrA*:A90V–*gyrB*:D461N (1)
AK	6	0	0	76	100.0	100.0	100.0	100.0	6	0	0	76	100.0	100.0	100.0	100.0	NA
ETO	3	0	5	74	100.0	93.67	93.90	37.50	8	0	0	74	100.0	100.0	100.0	100.0	*fabG1*:−15C/T (4), *fabG1*:−8T/A (1)
KM	7	0	0	75	100.0	100.0	100.0	100.0	7	0	0	75	100.0	100.0	100.0	100.0	NA
SM	32	3	8	39	91.43	82.98	86.59	80.00	40	3	0	39	93.02	100.0	96.34	100.0	*rpsL*:K43R (3), *rpsL*:K88R (1), *rrs*:514A/C (3), *rrs*:517C/T (1)
Total	253	15	50	502	94.40	90.94	92.07	83.50	303	15	0	502	95.28	100.0	98.17	100.0	

### Comparison between cfDAS-based mDST and Xpert MTB/RIF assay

We compared cfDAS-based mDST for RIF with the Xpert MTB/RIF assay in 113 specimens. The results showed that 111 specimens (98.23%) had consistent assay results, while two specimens (S001 and S494) were positive in Xpert MTB/RIF assay but negative in cfDAS-based mDST (Table S40). S001 showed a low-frequency mutation (S450L, 1.15% AF) in the *rpoB* gene in the cfDAS data and thus failed to pass the cutoff of 10% AF for mDST prediction. In S494, no mutations were detected in the 81-bp RIF resistance-determining region by cfDAS. We further compared the consistent results of cfDAS-based mDST and Xpert MTB/RIF with the pDST data, and found that 18 out of the 111 specimens showed inconsistent results. This result further indicates that culture indeed leads to bias in drug resistance identification.

## Discussion

Recent studies have provided evidence of diversity in drug resistance in colony-based MTBC populations from clinical specimens [24–26]. In addition, patients with MDR MTBC are more prone to acquiring resistance mutations [27]. The diversity spectrum of the bacterial population *in vivo* is influenced by intrinsic properties, adoption to the microenvironment of the host’s local niche, and the course of therapy [28]. However, the heterogeneity of mutations in current TB molecular diagnosis is rarely addressed due to the lack of efficient tools for high-coverage assay directly from clinical specimens (see File S1 for Appendix discussion 1) [29]. The conventional qualitative amplification of known resistance genes cannot be easily applied to the resistance diagnosis of TB; however, genotyping of the TB population in clinical specimens remains essential.

Hybridization-based enrichment was adopted to directly detect mutations in sputum sequencing with 5 ng of initial sputum DNA [7]. This increases the operational cost, such as more DNA or higher count of bacteria. Targeted sequencing using Illumina [8] and Oxford Nanopore Technologies [9] has become an alternative method for mDST. Here, we designed a DRM panel for 20 anti-TB drugs based on a literature repository that showed significant differences in the MTBC population between culture-free and culture-dependent stages using the within-sample amplicon sequencing, Xpert MTB/RIF, and WGS of isolates (see File S1 for Appendix discussion 2) (Figure S4; Tables S41–S43). The detection capability of our panel was evaluated systematically, showing a limit of detection comparable to that reported in a previous study (100–1000 genomes) based on amplicon sequencing [8].

Data pairs of cdDAS and cdWGS showed high consistency in AFs and target loci, while comparisons between cfDAS and cdWGS showed significant discrepancies in them (see File S1 for Appendix discussion 3; Figure S5). The cfDAS data showed a higher heterogeneity score compared to cdWGS and cdDAS data (14.67 *vs.* 0.07 in average for cfDAS *vs.* cdWGS, *P* = 1.4E−07, *t*-test; 14.67 *vs.* 0.09 in average for cfDAS *vs.* cdDAS, *P* = 1.5E−07, *t*-test) (Figure S6). To further investigate the discrepancies between cfDAS and cdWGS, the DRMs were compared and divided into three categories: (1) all DRMs with increased AFs (12 of 37 samples), (2) all DRMs with decreased AFs (1 of 37 samples), and (3) DRMs with increased or decreased AFs (24 of 37 samples) in cultured isolates. The AF change of DRMs during culture may be related to fitness cost (*e.g.*, *rpoB*:S450L emerged together with the compensatory mutation *rpoC*:I491V in S001), medication use [*e.g.*, *gyrA*:D94G emerged with the fluoroquinolone (FQ) treatment in S295], and culture (*e.g.*, *rpoB*:S450L increased but *rpoB*:D435G decreased in S074, and *embB*:Q497K and *gyrA*:D94G decreased in S077) (Figure 5). In addition, the higher proportion of pre-MDR and MDR MTBC in the patients was more likely to carry minor resistance mutations during the course of infection. Heteroresistance is common in the development of DR MTBC isolates [30], and was higher in cfDAS data than in cdWGS and cdDAS data (5.61 *vs.* 0.24 in average for cfDAS *vs.* cdWGS, *P* = 1.4E−08, *t*-test; 5.61 *vs.* 0.25 in average for cfDAS *vs.* cdDAS, *P* = 1.5E−08, *t*-test) (Figure S7). In addition, heterogeneity and heteroresistance were higher in bronchoalveolar lavage fluid (BALF) than in sputum in cfDAS data (Figure S8). These results indicate that the resistance profile of culture-free specimens is more complicated than that of culture-based isolates while BALF is more complicated than sputum in culture-free specimens.

Compared with the cdWGS data, 50 of 820 (6.10%) inconsistent mDST results due to 76 of 284 (26.76%) DRMs were found in the cfDAS data. For RIF, mDST results were consistent in culture-free samples (cfDAS and Xpert MTB/RIF) if two samples with low-frequency mutations or no mutations were excluded from the cfDAS data. Compared with the culture-dependent pDST data for RIF, 18 of 111 specimens (16.22%) showed inconsistencies in the cfDAS data, possibly due to low-level resistant mutations/disputed mutations (7 specimens) [31], AF differences (2 specimens), mutation changes (1 specimen), and time delay to positive pDST [32]. Compared with the cdDAS data, two specimens exhibited inconsistent RIF resistance predictions in the cfDAS data, possibly due to differences between culture-free and culture-dependent stages. Moreover, 33.33% (30/90) of the specimens with consistent RIF susceptibility between cfDAS and cdDAS data had different AFs (14 specimens, 15.56%) or different mutations (16 specimens, 17.78%).

Our study has several limitations. First, as a prospective single-center study focusing on culture-free detection of DRMs, the number of enrolled MTBC clinical specimens was limited to 115, and the sample size used in comparisons among different platforms was further reduced due to the failure of bacterial culture. Nevertheless, 87.83% of the specimens were RR, and our study highlighted the advantages of the cfDAS assay in the current clinical laboratory system, making further large-scale studies possible. Second, the pDST in this study covered only 12 drugs, and several important drugs (*e.g.*, pyrazinamide, prothionamide, and clofazimin) were not included. Nonetheless, comparisons between cdDAS- or cdWGS-based mDST and the corresponding pDST revealed good consistency. In addition, the *in silico* mDST predictions within the amplicon regions for several datasets were highly consistent with the cdWGS-based mDST results for 13 drugs. Third, the panel did not contain markers for distinguishing between MTBC and non-tuberculosis mycobacterium (NTM) species. Our panel was initially designed as a secondary clinical assay tool for high-potential MDR or extensively drug-resistant (XDR) patients. Numerous point-of-care testing methods, represented by Xpert, still have incomparable advantages in terms of speed and ease of use for primary screening of TB. Nevertheless, our study provides a sequencing-based resistance detection method that performs comparably to current clinical practices. It can be used to guide clinical medication and drug resistance monitoring. More importantly, this method can be used to study the discrepancies between clinical specimens and cultured isolates on a large scale, which has been ignored in previous studies owing to the lack of appropriate methods. Long-term accumulation of paired cfDAS and cdWGS data can enrich the TB resistance knowledge base, which will sustainably adjust and upgrade the target amplicon panel.

## Materials and methods

### Sample collection and processing

From November 2020 to April 2021, 115 clinical specimens were collected from TB patients with MTBC infection at the Third People’s Hospital of Shenzhen, Shenzhen, Guangdong Province, China. Several types of specimens were included in this study, including BALF, sputum, and others (*i.e.*, pleural fluid, pus, urine, and tissue). Each specimen was processed as follows: (1) the clinical specimen was initially tested with Xpert MTB/RIF assay (Cepheid, Sunnyvale, CA), and DNA extracted from the clinical specimen using the cetyltrimethylammonium bromide (CTAB) method was used for DAS [33]; (2) the cultured isolates of the clinical specimen were used for pDST using TREK Sensititre MYCOTB plates (TREK Diagnostic Systems, Oakwood, OH) for 12 anti-TB drugs; and (3) DNA extracted from the cultured isolates was used for DAS and WGS on the Illumina HiSeq2000 platform (Illumina, San Diego, CA).

### Sequencing data analysis

Paired-end reads of 150 bp were generated for each sample with average coverages of 17,919×, 43,651×, and 273× for cfDAS, cdDAS, and cdWGS data, respectively (Table S44). Adapter sequences and low-quality reads of raw sequencing data were filtered out using Trimmomatic (v0.39) with the parameters “ILLUMINACLIP:TruSeq3-PE.fa:2:30:15 LEADING:20 TRAILING:20 SLIDINGWINDOW:4:20 MINLEN:40”. Bases with quality scores below 20 were removed, and reads less than 40 bp were discarded. The filtered reads were aligned to the *M. tuberculosis* H37Rv genome (NC_000962.3) using BWA-MEM (v0.75a-r405). SNPs were identified using an in-house Perl script, bam2vcf, and the Genome Analysis Toolkit (GATK, v4.1.4.1). The Perl script bam2vcf detects variants using BAM files with strict filtering criteria to minimize the effects of contamination and coinfection. Specifically, mapping reads were filtered out if they met any of the following criteria: mapping loci outside of the targeted amplicon regions, mismatch rate ≥ 5%, mapping identity < 90%, or mapping quality (MAPQ) < 30. Variants were filtered out if allelic depth < 3× or minor AF < 1%. Comparing with WGS, DAS has unique data processing steps, including filtering sequencing data based on primer sequences and disabling fragment length filter. For consistency, SNPs with allelic depth < 3× were removed in the GATK calling.

### Panel design

Amplicon regions were collected and manually curated from a comprehensive literature review (Table S1). A multiplex PCR-based sequencing panel was designed to detect the aforementioned loci with a total length of 28,395 bp across 128 amplicons, which were divided into two pooled mixes for Illumina sequencing (see File S1 for Appendix method 1) (Table S45). Amplification efficiency, sequencing depth, limit of detection, and variant identification were optimized for both cultured isolates and clinical specimens (see File S1 for Appendix method 2).

### Xpert MTB/RIF-based mDST, acid-fast bacilli detection, MTBC culture, and pDST

The Xpert MTB/RIF assay was performed to detect RR-TB according to the manufacturer’s instructions for the GeneXpert machine (Cepheid, Sunnyvale, CA) (see File S1 for Appendix method 3). Direct smears for acid-fast bacilli were analyzed using traditional optical microscopy. Smear-positive specimens were cultured in Mycobacterial Growth Indicator Tubes (Becton Dickinson, Franklin, NJ) (see File S1 for Appendix method 4). Positive cultures were confirmed for mycobacteria by Ziehl–Neelsen staining. Species identification was performed using a commercial MPB64 monoclonal antibody assay (Genesis, Hangzhou, China). Indirect pDST was performed using MYCOTB plates (Thermo Fisher Scientific, Waltham, MA) for 12 anti-TB drugs (see File S1 for Appendix method 5). The minimum inhibitory concentration (MIC) was independently determined by two trained technicians using a Vizion Digital MIC Viewing System (Thermo Fisher Scientific) in accordance with the guidelines of the Clinical and Laboratory Standards Institute [34]. All pDSTs were performed twice for each isolate.

### Sequencing-based mDST

Variants with minimum AF ≥ 10% and minimum alternative allelic depth ≥ 3× were used for genotype-based mDST. Here, two methods were developed for mDST. The first method was based on known RDMs (referred to as the RDM method) (see File S1 for Appendix method 6). The second method was based on RF model built by the randomForest package in R [35] using the PATRIC data [36] as training data. This model was validated with three datasets: MTBC_A [19], MTBC_B [19], and WGS_110 data [20] (see File S1 for Appendix method 7). In addition, Mykrobe software (v0.9.0) was used for WGS-based mDST [19].

### Analyses of AF similarity, heterogeneity, and heteroresistance

AF similarity (AFS) refers to the consistency of AF at the same locus between two samples (AF1 and AF2) and was calculated using the formula: AFS = 1 − |AF1−AF2|. Heterogeneity is the coexistence of two alleles at a single locus, and the heterogeneity score (H) was calculated using the formula: H = 0.5 − |AF − 0.5|. Heteroresistance is defined as the presence of two or more mycobacterial populations with different drug susceptibilities. Here, we assigned a DRM as a heteroresistant mutation when its AF was ≥ 0.05 and < 0.95.

### Statistical analysis

The performance of mDST was evaluated using overall accuracy, sensitivity, and specificity. The 95% CI was calculated by the Rmisc package (v1.5.1). The Pearson’s correlation test, Pearson’s Chi-square test, and Student’s *t*-test were performed using R v3.6.3.

## Ethical statement

This study was approved by the Research Ethics Committee of the Third People’s Hospital of Shenzhen, China (Approval No. 2021-035-02). Written informed consents were obtained for all patients before study.

## Code availability

The code for variant identification based on BAM files is available at GitHub (https://github.com/liuwf-feige/bam2vcf). The code has also been submitted to BioCode at the National Genomics Data Center (NGDC), Beijing Institute of Genomics (BIG), Chinese Academy of Sciences (CAS) / China National Center for Bioinformation (CNCB) (BioCode: BT007389), which is publicly accessible at https://ngdc.cncb.ac.cn/biocode/tools/BT007389.

## Supplementary Material

qzae046_Supplementary_Data

## Data Availability

Raw sequencing data have been deposited in the Genome Sequence Archive [37] at the NGDC, BIG, CAS / CNCB (GSA: CRA013987 for cfDAS data, CRA013993 for cdWGS data, and CRA013994 for cdDAS data), and are publicly accessible at https://ngdc.cncb.ac.cn/gsa.

## CRediT author statement

**Jiuxin Qu:** Conceptualization, Supervision, Project administration, Funding acquisition. **Wanfei Liu:** Methodology, Software, Data curation, Investigation, Formal analysis, Validation, Visualization, Writing – original draft, Writing – review & editing. **Shuyan Chen:** Resources, Investigation, Formal analysis, Validation. **Chi Wu:** Resources, Investigation, Formal analysis, Validation. **Wenjie Lai:** Resources, Investigation. **Rui Qin:** Resources, Investigation. **Feidi Ye:** Resources, Investigation. **Yuanchun Li:** Resources, Investigation. **Liang Fu:** Resources. **Guofang Deng:** Resources. **Lei Liu:** Resources. **Qiang Lin:** Conceptualization, Supervision, Data curation, Formal analysis, Visualization, Writing – original draft, Writing – review & editing. **Peng Cui:** Conceptualization, Supervision, Project administration, Writing – review & editing. All authors have read and approved the final manuscript.
