# Supplementary material for: Deep Amplicon Sequencing Reveals Culture-dependent Clonal Selection of Mycobacterium tuberculosis in Clinical Samples
Source: Genomics Proteomics Bioinformatics. 2024 Jun 13;22(6):qzae046. doi: 10.1093/gpbjnl/qzae046 (PMC11978391; doi:10.1093/gpbjnl/qzae046)
Supplement: qzae046_Supplementary_Data [file qzae046_supplementary_data.zip › supplementary material captions.docx]

**Supplementary material**

**File S1 Supplementary methods**

**Figure S1 Circular display of TB drug resistant mutations in our panel**

Drug, gene, mutation, and physical map are displayed from inside to outside. Each drug is represented by one color [BDQ in RGB (102,102,0), CFZ in RGB (153,153,30), CM in RGB (204,0,0), CS in RGB (255,0,0), EMB in RGB (255,204,204), ETO in RGB (255,153,0), INH in RGB (255,204,0), KM in RGB (255,255,0), LZD in RGB (0,255,0), LFX/MFX in RGB (53,128,0), PAS in RGB (0,0,204), PMD in RGB (102,153,255), PTO in RGB (153,204,255), PZA in RGB (0,255,255), RFB/RFP in RGB (153,0,204), and SM in RGB (204,51,255)]. *gidB* and *rrs* are common target for AK/CM/KM/SM, *whiB7* is common target for AK/KM, *Rv0678* is common target for BDQ/CFZ, *ddn* is common target for DLM/PMD, *ethA* is common target for ETO/PTO, *fabG1* is common for ETO/INH, *inhA* is common target for INH/PTO, *gyrA* and *gyrB* are common target for LFX/MFX, and *rpoB* is common target for RFB/RFP. BDQ, bedaquiline; RGB, red, green, & blue; CFZ, clofazimine; CM, caperomycin; CS, cycloserine; LFX, levofloxacin; MFX, moxifloxacin; PAS, paraaminosalicylic acid; PTO, protionamide; PZA, pyrazinamide, LZD, linezolid; DLM, delamanid; PMD, pretonamid.

**Figure S2 The sequencing coverage of DAS data in three rounds of tests**

A01 to A03 are for amplification specificity and efficiency, B01 to B03 are for detection capability, and C01 to C03 are for amplification optimization. The horizontal axis indicates the drug resistant mutations in coordinate order, and the vertical axis indicates the log_10_ of sequence coverage (read depth).

**Figure S3 A Venn diagram showing the difference of drug susceptibility among three molecular DST methods**

A total of 574 phenotypes in 7 drugs were compared among RDM, Mykrobe, and random forest methods. RDM, resistance-determining mutation.

**Figure S4 Evaluation of variant identification among our pipeline (bam2vcf), VarScan2, and GATK using Illumina whole exome data HG002**

GATK, Genome Analysis Toolkit.

**Figure S5 The allele frequency change of drug resistant mutations and compensatory mutations between cfDAS and cdWGS data in 37 samples**

The horizontal axis indicates data types (1 stands for cfDAS and 2 stands for cdWGS) and the vertical axis indicates allele frequency.

**Figure S6 The heterogeneity comparison among cfDAS, cdDAS, and cdWGS data**

**Figure S7 The heteroresistance comparison among cfDAS, cdDAS, and cdWGS data**

**Figure S8 The heterogeneity and heteroresistance comparison between BALF and sputum in cfDAS, cdDAS and cdWGS data**

BALF, bronchoalveolar lavage fluid.

**Table S1 MTBC drug resistant mutations in our panel**

**Table S2 DAS for amplification specificity and efficiency, detection capability, and amplification optimization**

**Table S3 DAS for minimum limit of detection**

**Table S4 DAS for variant identification comparing with initial DNA sample**

**Table S5 pDST for PATRIC data**

**Table S6** **mDST based on RDM method in our panel region for PATRIC data**

**Table S7 Comparing pDST with mDST based on RDM method in our panel region for PATRIC data**

**Table S8 pDST for MTBC_A data**

**Table S9 mDST based on RDM method in our panel region for MTBC_A data**

**Table S10 Comparing pDST with mDST based on RDM method in our panel region for MTBC_A data**

**Table S11 Comparing pDST with** **mDST based on Mykrobe software for MTBC_A data**

**Table S12 pDST for MTBC_B data**

**Table S13 mDST based on RDM method in our panel region for MTBC_B data**

**Table S14 Comparing** **pDST with mDST based on RDM method in our panel region for MTBC_B data**

**Table S15 Comparing pDST with mDST based on Mykrobe software for MTBC_B data (obtained from article)**

**Table S16 pDST for WGS_110 data**

**Table S17 mDST based on RDM method in our panel region for WGS_110 data**

**Table S18 Comparing pDST with mDST based on RDM method in our panel region for WGS_110 data**

**Table S19 Comparing pDST with mDST obtained from article for WGS_110 data**

**Table S20 mDST based on random forest models in our panel region for PATRIC data**

**Table S21 Comparing pDST with** **mDST based on random forest models in our panel region for PATRIC data**

**Table S22 mDST based on random forest models in our panel region for MTBC_A data**

**Table S23 Comparing pDST with mDST based on random forest models in our panel region for MTBC_A data**

**Table S24 mDST based on random forest models in our panel region for MTBC_B data**

**Table S25 Comparing pDST with mDST based on random forest models in our panel region for MTBC_B data**

**Table S26 mDST based on random forest models in our panel region for WGS_110 data**

**Table S27 Comparing** **pDST with** **mDST based on random forest models in our panel region for WGS_110 data**

**Table S28 pDST for culture-dependent 82 clinical samples**

**Table S29 mDST based on RDM method in our panel region for cdWGS data of 82 clinical samples**

**Table S30 mDST based on random forest models in our panel region for cdWGS data of 82 clinical samples**

**Table S31 mDST based on Mykrobe software for cdWGS data of 82 clinical samples**

**Table S32 Comparing pDST with mDST based on RDM method in our panel region for cdWGS data of 82 clinical samples**

**Table S33 Comparing pDST with mDST based on random forest models in our panel region for cdWGS data of 82 clinical samples**

**Table S34 Comparing** **pDST with** **mDST based on Mykrobe software for cdWGS data of 82 clinical samples**

**Table S35 Different loci among cfDAS, cdDAS, and cdWGS data of 82 clinical samples**

**Table S36 mDST based on RDM method in our panel region for cfDAS data of 82 clinical samples**

**Table S37 Comparing pDST with mDST based on RDM method in our panel region for cfDAS data of 82 clinical samples**

**Table S38 mDST based on RDM method in our panel region for cdDAS data of 82 clinical samples**

**Table S39 Comparing pDST with mDST based on RDM method in our panel region for cdDAS data of 82 clinical samples**

**Table S40** **Rifampicin susceptibility test in culture-free (Xpert MTB/RIF and cfDAS) and culture-dependant stages (pDST, cdDAS, and cdWGS)**

**Table S41 Evaluation of variant identification using different cutoff value of alternative allele frequency in Illumina whole exome data HG002 (GIAB) among our pipeline (bam2vcf), VarScan2, and GATK**

**Table S42 Evaluation of variant identification using different cutoff value of total depth with alternative allele frequency >= 0.05 in Illumina whole exome data HG002 (GIAB) among our pipeline (bam2vcf), VarScan2, and GATK**

**Table S43 Evaluation of variant identification using different cutoff value of alternative allele depth with alternative allele frequency >= 0.05 and total depth >= 5X in Illumina whole exome data HG002 (GIAB) among our pipeline (bam2vcf), VarScan2, and GATK**

**Table S44 Statistics of cfDAS, cdDAS, and cdWGS data**

**Table S45 Amplification primers in our panel**
