# Supplementary figures and images for: Deep Amplicon Sequencing Reveals Culture-dependent Clonal Selection of Mycobacterium tuberculosis in Clinical Samples
Source: Genomics Proteomics Bioinformatics. 2024 Jun 13;22(6):qzae046. doi: 10.1093/gpbjnl/qzae046 (PMC11978391; doi:10.1093/gpbjnl/qzae046)

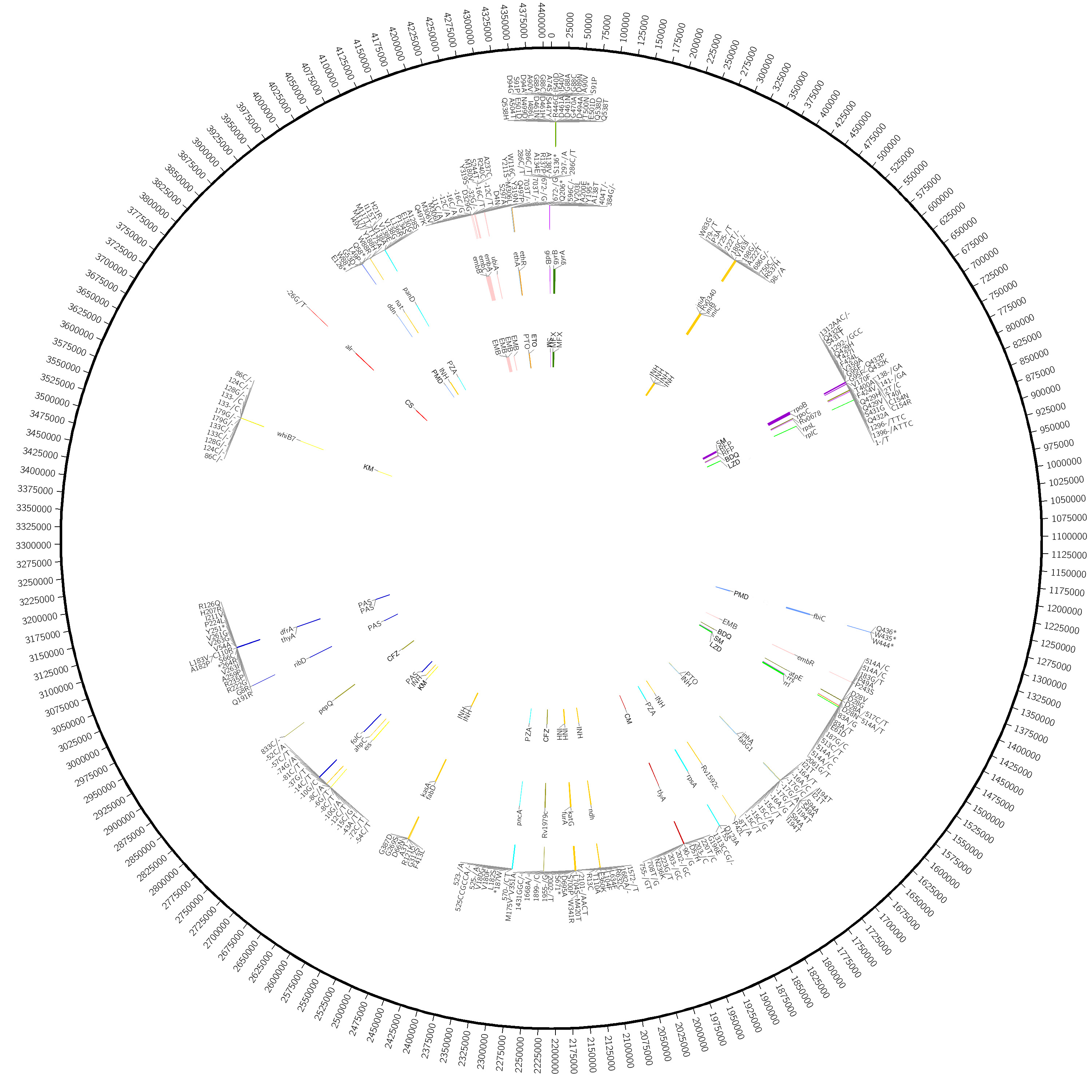

Supplement: qzae046_Supplementary_Data [file qzae046_supplementary_data.zip › Supplementary Figure 1.jpg]

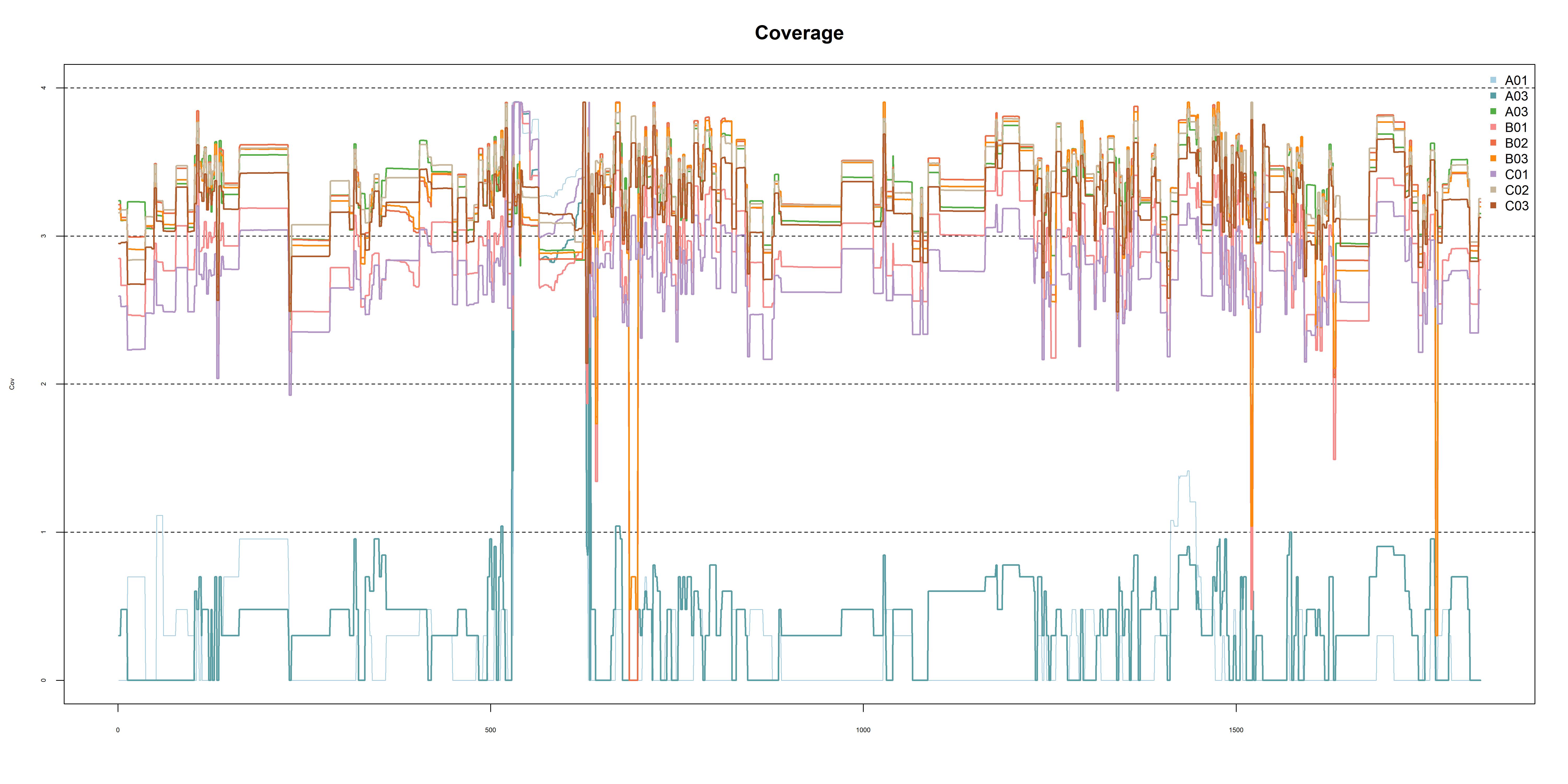

Supplement: qzae046_Supplementary_Data [file qzae046_supplementary_data.zip › Supplementary Figure 2.jpeg]

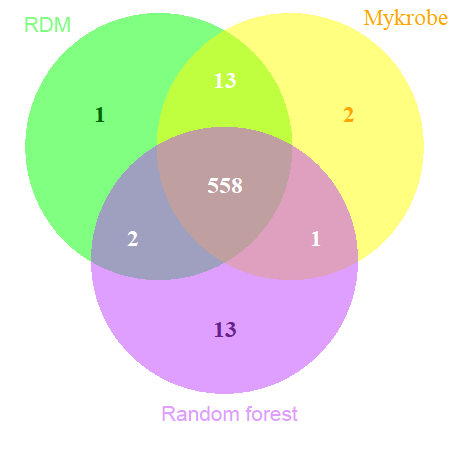

Supplement: qzae046_Supplementary_Data [file qzae046_supplementary_data.zip › Supplementary Figure 3.png]

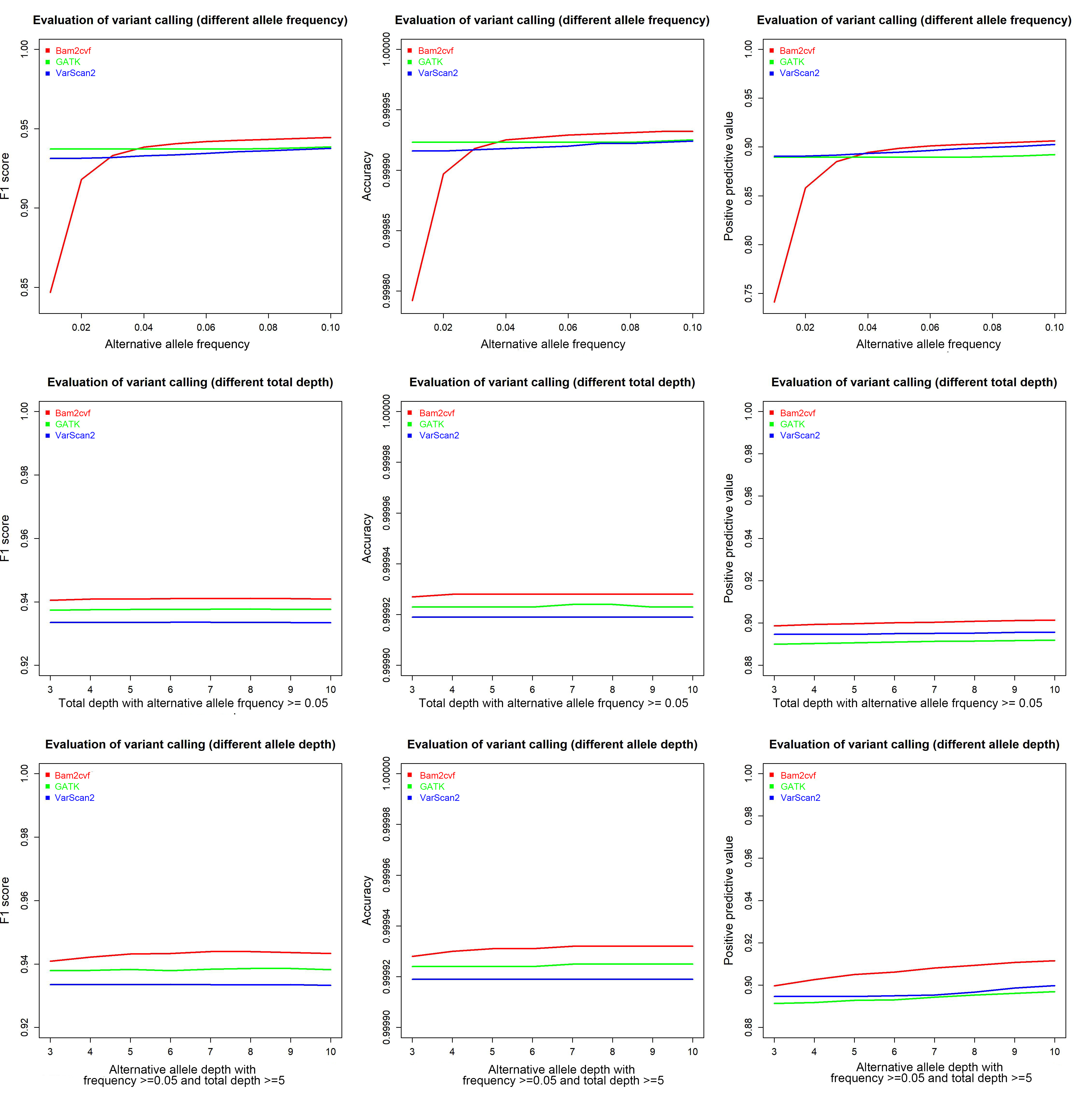

Supplement: qzae046_Supplementary_Data [file qzae046_supplementary_data.zip › Supplementary Figure 4.jpg]

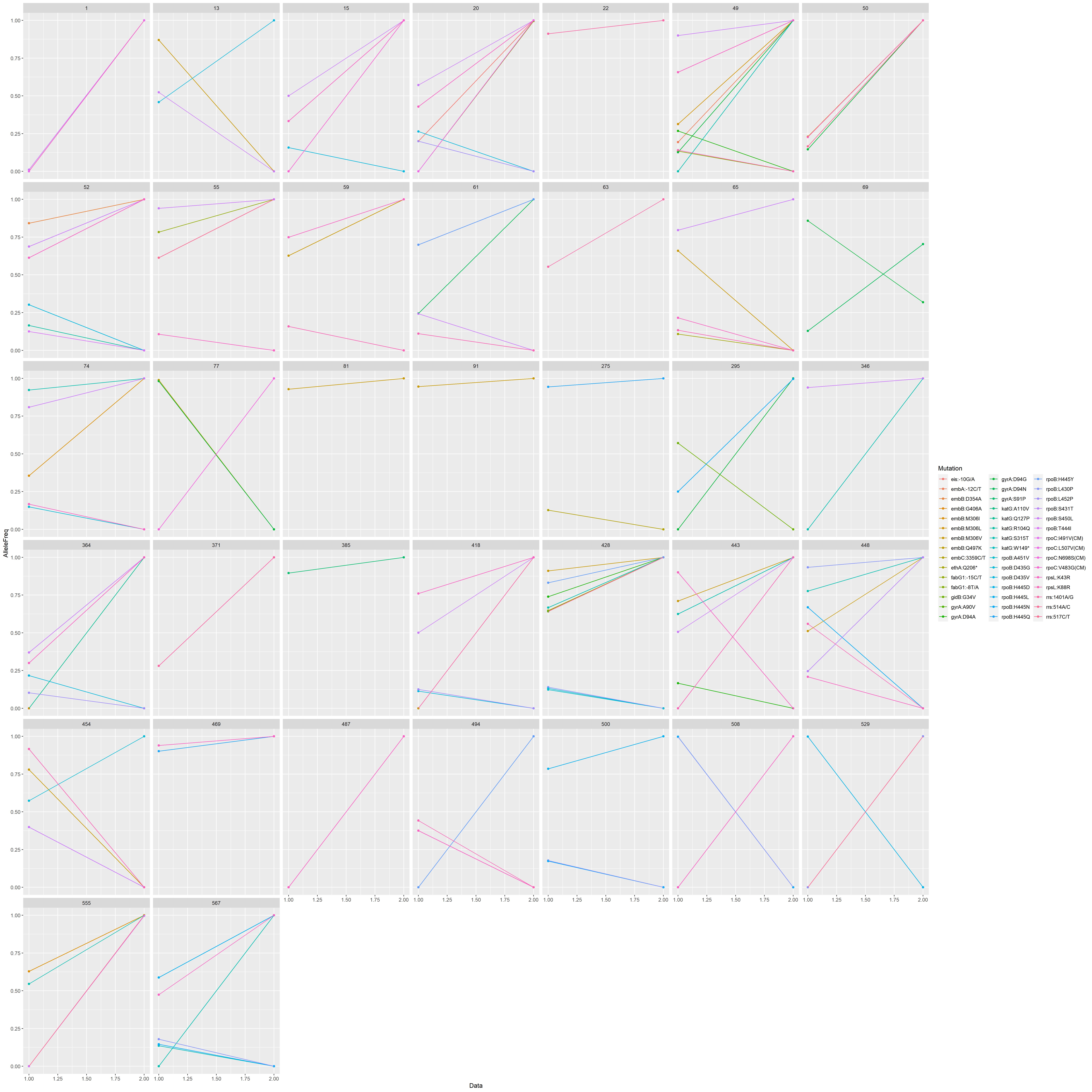

Supplement: qzae046_Supplementary_Data [file qzae046_supplementary_data.zip › Supplementary Figure 5.jpeg]

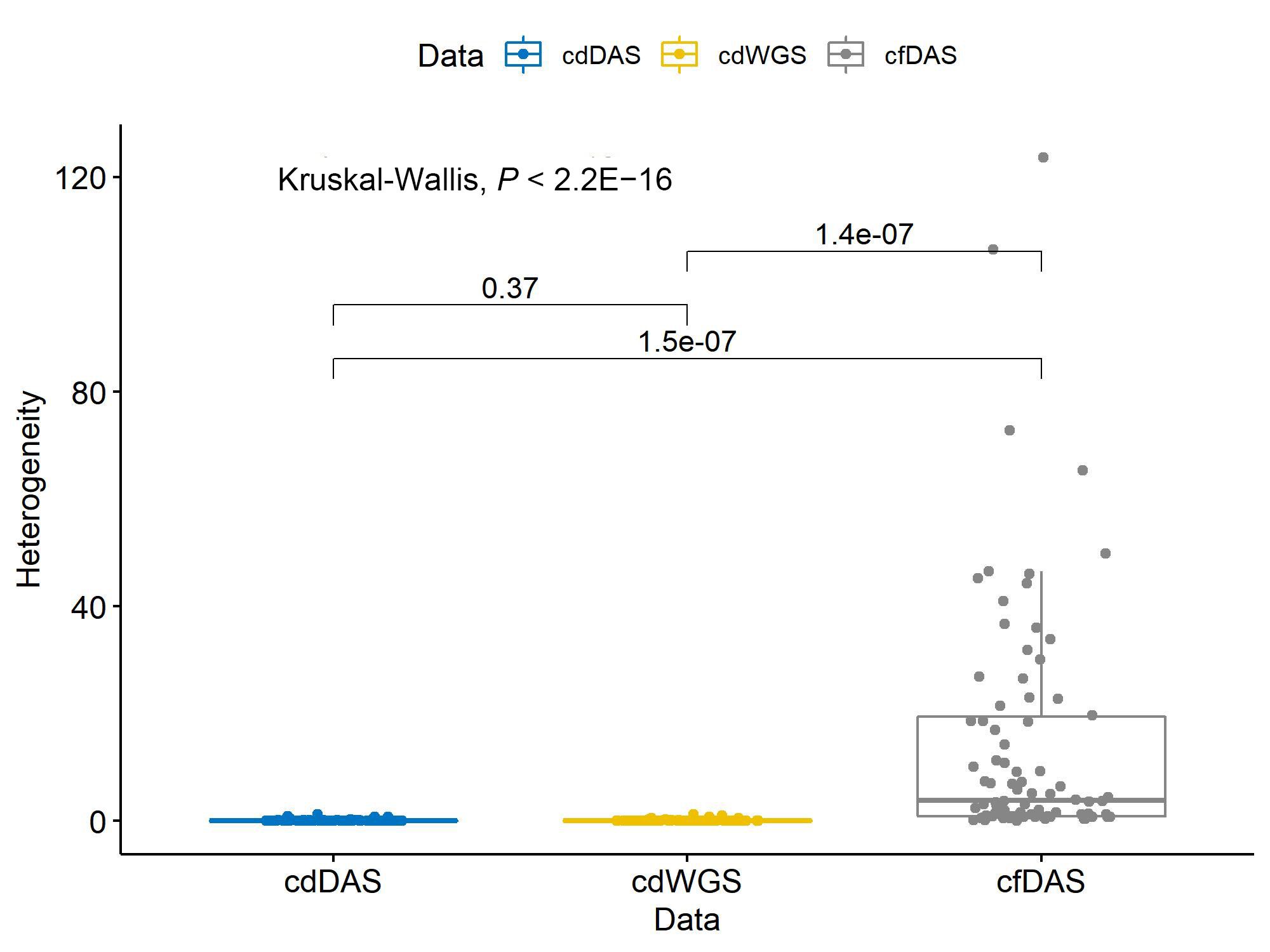

Supplement: qzae046_Supplementary_Data [file qzae046_supplementary_data.zip › Supplementary Figure 6.jpg]

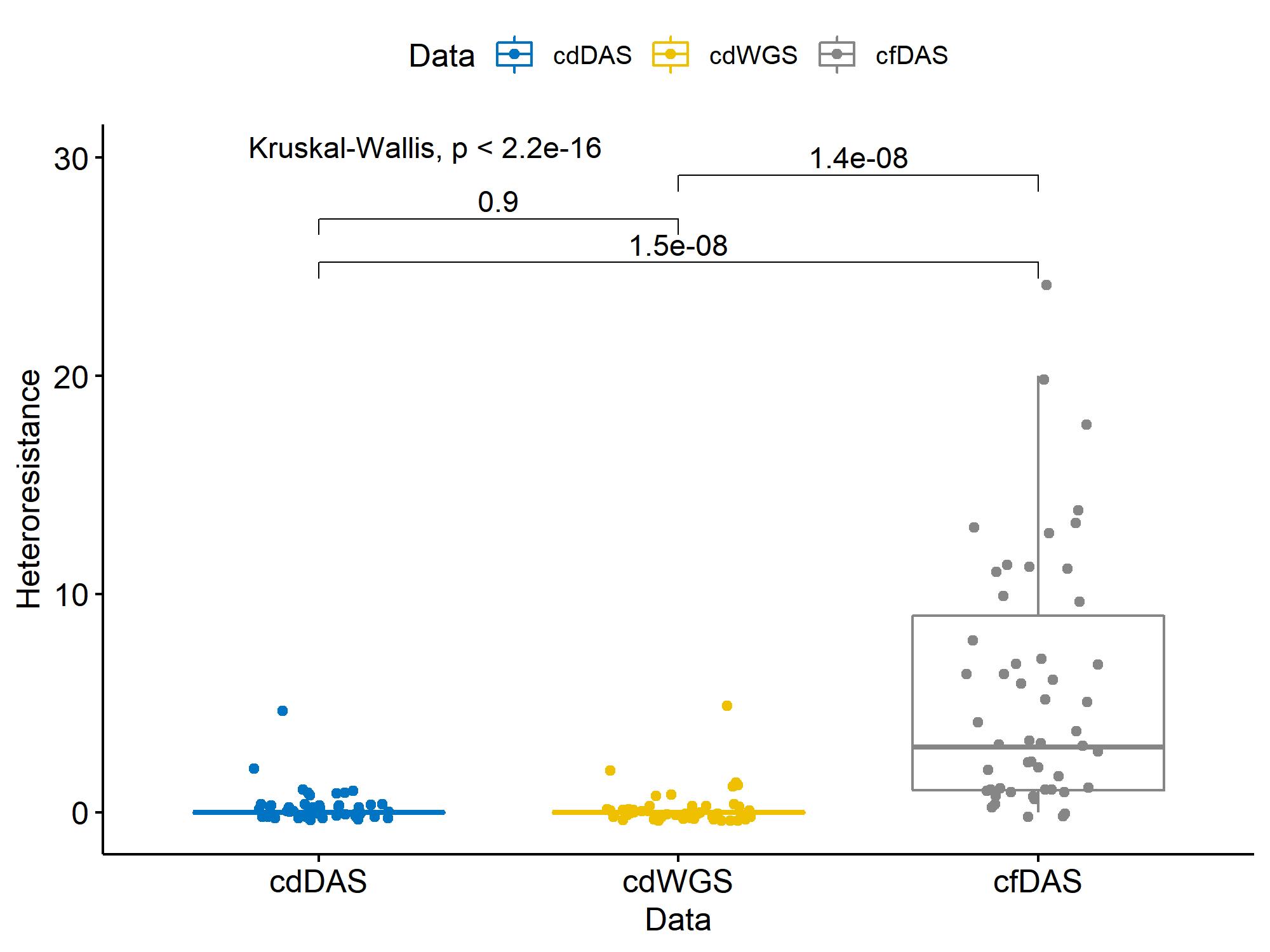

Supplement: qzae046_Supplementary_Data [file qzae046_supplementary_data.zip › Supplementary Figure 7.jpeg]

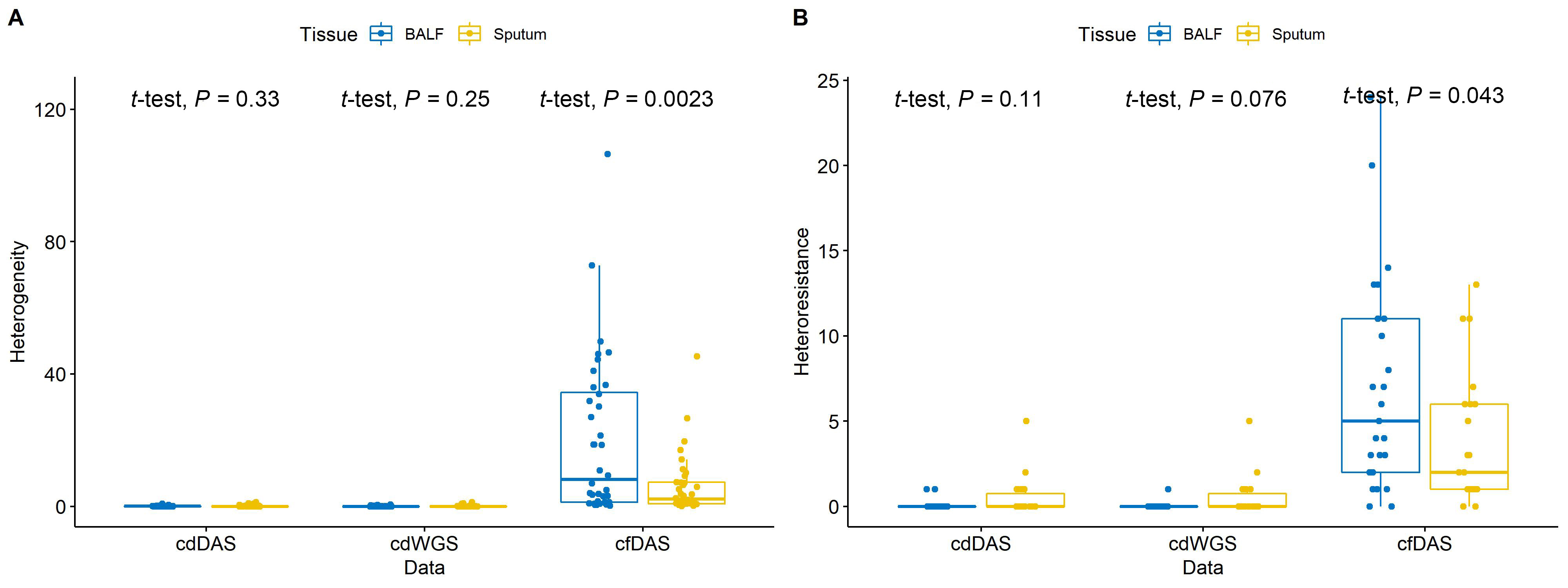

Supplement: qzae046_Supplementary_Data [file qzae046_supplementary_data.zip › Supplementary Figure 8.jpg]
