# Supplementary material for: Deep Amplicon Sequencing Reveals Culture-dependent Clonal Selection of Mycobacterium tuberculosis in Clinical Samples
Source: Genomics Proteomics Bioinformatics. 2024 Jun 13;22(6):qzae046. doi: 10.1093/gpbjnl/qzae046 (PMC11978391; doi:10.1093/gpbjnl/qzae046)
Supplement: qzae046_Supplementary_Data [file qzae046_supplementary_data.zip › File S1.doc]

**File S1 Supplementary methods**

**Appendix result 1: drug resistance prediction**

Based on our newly obtained data and their corresponding phenotypic drug susceptibility testing (pDST), we predicted the drug resistance for 10 drugs [amikacin (AK), ethambutol (EMB), ethionamide (ETO), levofloxacin (LFX), isoniazid (INH), kanamycin (KM), moxifloxacin (MFX), rifabutin (RFB), rifampicin (RFP), and streptomycin (SM)] by the resistance-determining mutation (RDM) and random forest methods, then compared the *in silico* predictions to pDST. The overall accuracies of drug resistance prediction of our deep amplification sequencing (DAS) panel were 92.07% (94.40% sensitivity and 90.94% specificity) and 91.34% (91.29% sensitivity and 91.37% specificity) using the RDM and random forest methods, respectively. Next, we compared the drug resistance prediction ability of our panel with that of the Mykrobe software for our newly generated whole genome sequencing (WGS) data. The Mykrobe software is commonly used for WGS-based drug resistance prediction and the overall accuracy of drug resistance prediction was 92.16% (95.02% sensitivity and 90.62% specificity) for seven common drugs. In contrast, the overall accuracy of drug resistance prediction of our panel was 93.03% (96.10% sensitivity and 91.33% specificity) and 91.99% (92.54% sensitivity and 91.69% specificity) using the RDM and random forest methods, respectively, for seven common drugs.

**Appendix result 2: culture-free DAS and culture-dependent WGS comparison**

Culture-dependent WGS (cdWGS) is a high-throughput method that is currently used for molecular drug susceptibility testing (mDST) in clinics. Therefore, we performed a comparative study between our culture-free DAS (cfDAS) and cdWGS in 83 clinical specimens. We obtained 117 Gb and 139 Gb high-quality raw sequences, with average coverage of 17,919X and 273X on the targeted genomic region for each specimen for cfDAS and cdWGS, respectively. After alignment, 9060 and 1231 sequence variations [including single nucleotide polymorphisms (SNPs) and insertion–deletions (InDels)] were identified for cfDAS and cdWGS, respectively. Comparing the SNPs identified by cfDAS and cdWGS, 7336 SNPs showed difference in allele frequency (AF), using a cutoff minimum of 5% frequency change in all 83 specimens. Among these SNPs, 333 SNPs from 49 specimens belonged to drug-resistant (DR) mutations that were linked to 16 drugs, namely AK (36), caperomycin (CM) (36), KM (37), SM (33), bedaquiline (BDQ) (1), clofazimine (CFZ) (40), EMB (38), ETO (7), INH (25), LFX (23), MFX (23), paraaminosalicylic acid (PAS) (7), prothionamide (PTO) (10), pyrazinamide (PZA) (64), RFB (24), and RFP (54). We also performed cdDAS on 83 cultured isolates. As a result, 147 Gb of high-quality raw sequences were generated, with an average coverage of 43,651X on the targeted genomic region for each isolate. A total of 1147 SNPs and 92 InDels were identified within the target genomic regions.

**Appendix result 3: mDST and pDST comparison**

Based on DR mutations identified by cfDAS and cdWGS data, we performed mDST for 82 specimens using the RDM method and compared them with the corresponding pDST of ten drugs. The DR mutations with genotype difference in cfDAS and cdWGS caused discrepancy of mDST. For example, specimen S001 is pan-susceptible based on mDST in cfDAS data, but pre-extensive drug resistance based on mDST in cdWGS data (EMB:*embB*:M306V, LFX/MFX:*gyrA*:A90V, INH:*katG*:S315T, RFB/RFP:*rpoB*:S450L, and SM:*rpsL*:K43R). Another example is specimen S074, which contains mutations with different AFs (EMB:*embB*:M306I, 35.48% in cfDAS and 100% in cdWGS; INH:*katG*:S315T, 92.31% in cfDAS and 100% in cdWGS; and RFB/RFP:*rpoB*:S450L, 80.85% in cfDAS and 100% in cdWGS) and mutations with different genotypes (RFP:*rpoB*:D435G, 14.89% in cfDAS and 0% in cdWGS; SM:*rpsL*:K43R, 16.67% in cfDAS and 0% in cdWGS).

**Appendix discussion 1: disadvantages of culture**

Infections caused by clinically important mycobacteria are an important public health problem worldwide, primarily due to the emergence of drug resistance . pDST is currently the gold standard for identifying drug resistance in clinics. However, its accuracy is influenced by bacterial load , bacterial population , culture condition , and fitness cost caused by DR mutation . The WGS-based mDST has the same issue inherited from bacterial culture. Contrastingly, cfDAS directly detects DNA in clinical specimens without culturing and thus can accurately detect *Mycobacterium tuberculosis* complex (MTBC) at the population level. Tuberculosis (TB) resistance is derived from DNA mutations in diverse functional elements, such as metabolic genes, antibiotic-binding targets, or transcription factors, and compensatory mutations can mitigate the deleterious effects of the specific functions compromised by resistance mutations . Unlike other bacteria, pathogenic TB rarely acquires exogenous DNA, whether by mobile plasmid or by recombination between isolates . The regular qualitative amplification of known resistance genes cannot be easily applied to the resistance diagnosis of TB; however, genotyping of the TB population in clinical specimens is necessary.

**Appendix discussion 2: design of panel and bioinformatics analysis**

A total of 941 DR mutations in 47 genes were included, and the selected regions had approximate efficacy with WGS under the current resistance prediction method, making it the largest panel for targeted sequencing of MTBC at present. Multiplex polymerase chain reaction (PCR) was developed for simultaneous amplification, and DAS using Illumina was used for raw data generation. A bioinformatics pipeline was designed to process targeted sequencing and WGS data, including several filters to eliminate non-MTBC reads and obtain reliable variant identification. The key analysis steps were read filtering based on the read alignment and variant filtering according to the base quality and allele depth. Using Illumina whole-exome data of HG002 and its high-confidence variant calls from the Genome in a Bottle (GIAB) as benchmark data , variants identified by our pipeline (called bam2vcf), Genome Analysis Toolkit (GATK) , and VarScan2 were compared. The F1 score, accuracy, and positive predictive value were higher in bam2vcf than in GATK and VarScan2 when the alternative AF was ≥ 5% and alternative allele depth was ≥ 3X.

**Appendix discussion 3: comparison among cfDAS and cdWGS**

To further inspect the discrepancy between cfDAS and cdWGS, the DR mutations were compared and divided into three categories: (1) AF upregulated (12 of 37 samples, *e.g.*, S001), (2) AF downregulated (one of 37 samples, *e.g.*, S077), and (3) AF upregulated and downregulated (24 of 37 samples, *e.g.*, S074) in cultured isolates. The change of resistance mutations during culture may be related to fitness cost (for instance, *rpoB*:S450L emerged together with compensatory mutation *rpoC*:I491V in S001) , medication use (for instance, *gyrA*:D94G emerged with FQs in the current treatment in S295), and culture (for instance, *rpoB*:S450L increased but *rpoB*:D435G decreased in S074). In addition, the higher proportion of multidrug-resistant (MDR) and pre-MDR in the patients is more likely to carry minor resistance mutations during the course of infection .

**Appendix method 1: multiplex PCR-based DAS**

A multiplex PCR-based sequencing panel was designed to detect the target comprising 941 loci from 47 genes associated with 20 anti-TB drugs. The main drug catalogs included five first-line drugs (INH, RFP, RFB, EMB, and PZA) and 11 second-line drugs [LFX, MFX, linezolid (LZD), CFZ, AK, CM, KM, SM, ETO, PTO, and PAS]. The four remaining anti-TB drugs [cycloserine (CS), BDQ, delamanid (DLM), and pretomanid (PMD)] have only a few known DR mutations. The whole target was 28,395 bp length in 128 amplicons. The multiplex PCR products were amplified using sample index and sequencing adaptor of the Illumina platform (Illumina, San Diego, CA). All sequencing was performed on an Illumina HiSeq2000 sequencing system (Illumina, San Diego, CA) following the manufacturer’s standard protocol.

**Appendix method 2: DAS amplification efficiency, sequencing depth, limit of detection, and variant identification**

Four rounds of DAS tests were done for amplification efficiency, sequencing depth, and limit of detection. In the first round, comparing with sample from culture-dependent TB DNA with 91.02% reads mapped and more than 2000X sequencing depth, samples from two healthy people sputums only mapped 1.01% to 2.20% reads with 30X sequencing depth, which indicated that our panel had high amplification specificity. Meanwhile, 30X sequencing coverage of TB DNA sample was 97.32%, suggesting a high amplification sensitivity. Using 50 pg, 100 pg and 200 pg culture-dependent TB DNA as template, our panel can detect about 97% target region with at least 30X sequencing coverage (2180X average coverage) using less than 200 Mb sequencing data in the second round. After amplification optimization, our panel can achieve 99.37% to 100% 30X sequencing coverage for the target region in the third round. In the fourth round, the assay’s limit of detection was confirmed with the DNA extracted from culture isolate in gradient dilution (40 ng, 4 ng, 400 pg, 40 pg, and 4 pg). According to the comparison between the results of our panel assay and WGS, the panel can currently efficiently detect a minimum concentration of 0.4 pg/ul TB DNA with 10 μl volume with 99.75% accuracy of variant detection (4 pg DNA, about 1000 copies of TB genome).

**Appendix method 3: mDST by Xpert**

We used the Xpert® *Mycobacterium tuberculosis /* rifampicin (Xpert MTB/RIF) assay (Cepheid, Sunnyvale, CA) on specimens from patients with TB to detect rifampicin-resistant (RR)-TB. The procedure was performed according to the instructions of Xpert MTB/RIF: 1 ml specimen was added into a 50 ml Falcon tube (Fisher Scientific, Rockingham, NH) containing 3 ml GeneXpert reaction mix, vortexed, and then left to stand for 20 min. The clear reactant–sample mixture (2 ml) was added to the GeneXpert MTB/RIF cartridge and processed using the GeneXpert machine GeneXpert (Cepheid, Sunnyvale, CA). A direct smear for Acid Fast Bacilli (AFB) was analyzed using traditional optical microscopy.

**Appendix method 4: MTBC culture**

A smear-positive specimen was digested using the N-acetyl-L-cysteine-NaOH method for 15 min and then neutralized with sterile phosphate buffer (PBS, pH = 7.0). After centrifugation at 3000 × g for 15 min, the pellet was resuspended in 2 ml PBS buffer. The decontaminated specimen (0.5 ml) was cultured in Mycobacterial Growth Indicator Tubes (Becton Dickinson, Franklin, NJ).

**Appendix method 5: pDST**

Indirect drug susceptibility of the culture-positive MTBC isolates was performed using MYCOTB plates (Thermo Fisher Scientific, Waltham, MA) for 12 drugs (INH, RFP, EMB, SM, ofloxacin, MFX, KM, AK, RFB, CS, ETO, and PAS). All steps were conducted strictly according to the manufacturer’s instructions by trained technicians at the Department of Clinical Laboratory of the National Clinical Research Center for Infectious Diseases. Briefly, a McFarland suspension of MTBC (0.5 ml) was prepared using an ultrasonic milling instrument (TB Healthcare, Guangdong, China) from logarithmic growth colonies. The suspensions were diluted 100-fold by adding 100 ml of the 0.5 McFarland suspension to 10 ml Middlebrook 7H9 Broth (Thermo Fisher Scientific, Waltham, MA) with 10% OADC (BD, Franklin Lakes, NJ). Aliquots of 100 ml of standard 1.5 × 10^5^ CFU/ml inoculum were distributed to each well using a semiautomated Sensititre auto-inoculator (Thermo Fisher Scientific, Waltham, MA). All the plates were sealed with adhesive membranes and incubated at 37°C in 5% CO_2_. After incubation for 10–21 days, the DST results were read separately by two trained technicians using a Sensititre Vizion Digital MIC Viewing System. The minimum inhibitory concentration (MIC) was defined as the lowest antibiotic concentration that inhibited visible growth of a microorganism. All results were valid if there were no skipped wells (bacterial growth was observed in wells containing higher concentrations of anti-TB drug, but not in wells containing lower concentrations of anti-TB drug), no contamination, and both positive-control wells had distinct bacterial growth. H37Rv (ATCC 27294) was used as a pan-susceptibility control for each batch of DST.

**Appendix method 6: RDM method for mDST**

An in-house Perl script was developed for anti-TB drug resistance prediction based on the resistance mutation panel and variant calling result in Genomic Variant Call Format (GVCF) format. The resistance mutation panel was built by manually curated gene mutations relating to drug resistance from a comprehensive literature review. The genotype of sample was obtained according to the variant calling result in targeted region using the following criteria: (1) for variant, assigning alternative allele as genotype if the minimum alternative allele depth >= 3X and the minimum alternative AF >= 10%, otherwise, designating reference allele as genotype if the minimum reference allele depth >= 3X; and (2) for consensus site, assigning reference allele as genotype if the sequencing depth >= 3X. By mapping genotype to resistance mutation panel, drug resistance was obtained based on the following criteria: (1) the genotype is known resistance mutation; (2) the genotype can lead amino acid mutation causing drug resistance; (3) the genotype can lead frameshift mutation or amino acid mutation in *pncA* gene for INH resistance, and *Rv0678* gene for CFZ resistance; and (4) the genotype can lead frameshift mutation or premature stop codon in *ethA* gene for PTO resistance, and *pepQ* gene for CFZ resistance.

**Appendix method 7: random forest method for mDST**

Based on the Pathosystems Resource Integration Center (PATRIC) data, random forest models were built by randomForest package in R. First, sequence alignment was obtained by comparing DR genes of *Mycobacterium tuberculosis* H37Rv with genome sequence of PATRIC data using BLASTN software. Then, the genotype of each isolate was obtained according to the sequence alignment. Third, odds ratio (OR) value was calculated for each variant by comparing the number of different genotypes between resistant and susceptible groups based on the result of pDST, and the genotype with OR value >= 2 was identified as potential DR mutations. Fourth, random forest model was constructed using genotypes of potential DR mutations identified in the previous step and known DR mutations. In this step, we first transformed genotype and pDST data into readable data (A, C, G, T, insertion, deletion, and missing value were represented by 1, 2, 3, 4, 5, 6, and 0, respectively, for genotype data, and sensitivity and resistance were represented by 0 and 1, respectively, for pDST data) by randomForest package in R, then divided PATRIC data into training and testing data in a ratio of 7:3, next built random forest model using training data, and validated it using testing data. Because sample size of many drugs was small, we reconstructed random forest model for each drug with whole PATRIC data and validated it using MTBC_A, MTBC_B, and Wenhong Zhang data.

**References**

[1] Nimmo C, Brien K, Millard J, Grant AD, Padayatchi N, Pym AS, et al. Dynamics of within-host *Mycobacterium tuberculosis* diversity and heteroresistance during treatment. EBioMedicine 2020;55:102747.

[2] Palaci M, Dietze R, Hadad DJ, Ribeiro FKC, Peres RL, Vinhas SA, et al. Cavitary disease and quantitative sputum bacillary load in cases of pulmonary tuberculosis. J Clin Microbiol 2007;45:4064–6.

[3] Ejo M, Van Deun A, Nunn A, Meredith S, Ahmed S, Dalai D, et al. Effectiveness of GenoType MTBDR*sl* in excluding TB drug resistance in a clinical trial. Int J Tuberc Lung Dis 2021;25:839–45.

[4] McIvor A, Koornhof H, Kana BD. Relapse, re-infection and mixed infections in tuberculosis disease. Pathog Dis 2017;75:ftx020.

[5] Lawson L, Emenyonu N, Abdurrahman ST, Lawson JO, Uzoewulu GN, Sogaolu OM, et al. Comparison of *Mycobacterium tuberculosis* drug susceptibility using solid and liquid culture in Nigeria. BMC Res Notes 2013;6:215.

[6] Emane AKA, Guo X, Takiff HE, Liu S. Drug resistance, fitness and compensatory mutations in *Mycobacterium tuberculosis*. Tuberculosis 2021;129:102091.

[7] Cabibbe AM, Spitaleri A, Battaglia S, Colman RE, Suresh A, Uplekar S, et al. Application of targeted next-generation sequencing assay on a portable sequencing platform for culture-free detection of drug-resistant tuberculosis from clinical samples. J Clin Microbiol 2020;58:e00632-20.

[8] Deng Q, Meng J, Guan Y, Liu Y, Xiao C. Plasmid to generate *Mycobacteria* mutants. AMB Express 2018;8:13.

[9] Krusche P, Trigg L, Boutros PC, Mason CE, De La Vega FM, Moore BL, et al. Best practices for benchmarking germline small-variant calls in human genomes. Nat Biotechnol 2019;37:555–60.

[10] DePristo MA, Banks E, Poplin R, Garimella KV, Maguire JR, Hartl C, et al. A framework for variation discovery and genotyping using next-generation DNA sequencing data. Nat Genet 2011;43:491–8.

[11] Koboldt DC, Zhang Q, Larson DE, Shen D, McLellan MD, Lin L, et al. VarScan 2: somatic mutation and copy number alteration discovery in cancer by exome sequencing. Genome Res 2012;22:568–76.

[12] Comas I, Borrell S, Roetzer A, Rose G, Malla B, Kato-Maeda M, et al. Whole-genome sequencing of rifampicin-resistant *Mycobacterium tuberculosis* strains identifies compensatory mutations in RNA polymerase genes. Nat Genet 2012;44:106–10.

[13] Vargas R, Freschi L, Marin M, Epperson LE, Smith M, Oussenko I, et al. In-host population dynamics of *Mycobacterium tuberculosis* complex during active disease. Elife 2021;10:e61805.
